# Hepatoprotective effects of *Juglans regia* on carbon tetrachloride‐induced hepatotoxicity: In silico/in vivo approach

**DOI:** 10.1002/fsn3.4288

**Published:** 2024-06-18

**Authors:** Bipindra Pandey, Shankar Thapa, Atisammodavardhana Kaundinnyayana, Sushil Panta

**Affiliations:** ^1^ Department of Pharmacy Madan Bhandari Academy of Health Sciences Hetauda Nepal; ^2^ School of Health and Allied Sciences Pokhara University Pokhara Nepal

**Keywords:** carbon tetrachloride, hepatoprotective, *Juglans regia* L., molecular docking, serum biochemical parameters

## Abstract

*Juglans regia* L. is a well‐known therapeutic plant in Nepal, employed in traditional medicine for treating liver ailments. This study aimed to evaluate the in vivo and in silico liver‐protective effects of *J. regia* extract using a carbon tetrachloride (CCl_4_)‐induced hepatic damage rat model. Healthy male rats were randomly divided into six groups: normal control (distilled water 10 mL/kg), toxic control (distilled water 10 mL/kg), standard test (silymarin 100 mg/kg), and three groups receiving oral *J. regia* extracts (125, 250, and 500 mg/kg/day) for seven days. On the eighth day, carbon tetrachloride (CCl_4_) was administered intraperitoneally (i.p.) (1.5 mL/kg in 1:1 olive oil ratio for all groups, except the normal control). Rats were sacrificed on the ninth day, and blood was collected retro‐orbitally for liver blood injury tests and histopathological studies. Molecular docking was performed against cytochrome P450 2E1 (CYP450 2E1) enzyme for 16 selected phytoconstituents. *J. regia*, at doses of 125, 250, and 500 mg/kg, significantly reduced liver enzyme levels (alanine aminotransferase, alkaline phosphatase, direct bilirubin, and total bilirubin), while increasing serum albumin. Histological analysis revealed mitigation of carbon tetrachloride (CCl_4_)‐induced liver injury, reducing fatty degeneration and necrosis. Molecular docking supported the findings, with Beta‐sitosterol and Betulinic acid exhibiting the best binding affinity of −9.2 and −9.1 kcal/mol, respectively. In conclusion, result suggests that *J. regia* showed dose‐dependent hepatoprotective activity in CCl_4_‐induced hepatotoxicity and it could be utilized as a promising hepatoprotective agent. This study suggests the hepatoprotective potential of *J. regia* bark extracts, emphasizing the need for further clinical validation.

## Graphical abstract

This study evaluated the liver‐protective effects of *Juglans regia* L. extract using a carbon tetrachloride (CCl_4_)‐induced hepatic damage rat model, demonstrating significant reductions in liver enzyme levels and bilirubin, along with histological evidence of liver injury mitigation. Molecular docking analysis highlighted Beta‐sitosterol and Betulinic acid as potential bioactive compounds, suggesting the hepatoprotective potential of *J. regia* bark extracts and indicating the necessity for additional clinical validation.

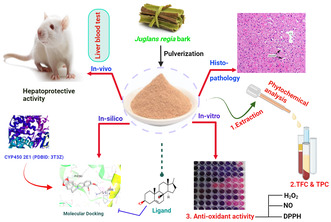

## 1 INTRODUCTION

The liver is an essential organ that plays a major role in the body's ability to maintain physiological homeostasis, fight disease, maintain metabolic processes, store vitamins, metabolize drugs, and protect the body from xenobiotics and toxic substances. It also secretes bile and is involved in the metabolism of biomolecules, which include proteins, carbohydrates, and fats (Real et al., 2019; Song et al., 2024; Ugwu & Suru, 2021).

Two million deaths per year are caused by liver disease, which also accounts for 4% of all deaths globally (1 out of every 25 deaths); men make up about two thirds of liver‐related deaths. Acute hepatitis accounts for a lesser percentage of deaths, with complications from cirrhosis and hepatocellular carcinoma being the main causes of death. The most prevalent causes of cirrhosis globally are alcohol consumption, nonalcoholic fatty liver disease (NAFLD), and viral hepatitis (Devarbhavi et al., 2023; Younossi et al., 2023). According to research, paracetamol overdose is linked to nearly 40% of drug‐associated liver problems (Cohen et al., 2024; Omotayo et al., 2015). For example, among the top 10 main causes of death globally are drug‐induced hepatic damage and chronic liver disorders, necessitating safe and efficient treatments (Pandey et al., 2023).

There are a lot of contemporary medications on the market, but only a small number of herbal remedies have been proven to help treat liver disease. These medications strengthen the liver's protective capabilities, restore damaged hepatic cells, and enhance liver effectiveness (Maqbool et al., 2019). Based on experimental verification, natural bioactive components generated from plant secondary metabolites have been announced as valuable alternatives for predicting and mitigating hepatotoxic effects and their chronic consequences (Pandey et al., 2023).

Natural products rich in antioxidants are utilized in traditional systems of medicine for treating liver disease due to its effectiveness and safety profile (Amin & Nagy, 2009; Pandey et al., 2013; Souid et al., 2024). Walnut fruits and leaves contain phenolic compounds, vitamins, and carotenoids that show antioxidant activity, so this prevents the attack from free harmful radicals and oxidative stress‐induced liver damage (Aydın et al., 2015). Walnut tree bark (*Juglans regia* L.) is a common medicinal plant known as ‘Okhar’ in Nepal, prescribed by the folk medicine systems to treat liver disease, which could be due to their anti‐inflammatory and blood‐purifying activities (Al‐Nadaf et al., 2024; Asha et al., 2010). *Juglans regia* L. (*J. regia*) bark was selected for the phytochemical analysis, antioxidant and in vivo/in silico hepatoprotective activity based on its traditional uses. A well‐established experimental rat model of hepatic damage caused by carbon tetrachloride (CCl_4_) is used to examine the hepatoprotective efficacy and basic molecular mechanisms of new medicines (El‐Beltagi et al., 2024; Kourkoumpetis & Sood, 2019; Yan et al., 2018). Additionally, molecular docking approach is used to establish the interaction of ligands with particular receptor. Overall, our study aim to evaluate the in vivo hepatoprotective activity of *Juglans regia* L. extract using a carbon tetrachloride‐induced hepatic damage rat model is confirmed by in silico studies. Furthermore, we have selected the main phytochemicals of *Juglans regia* L. bark as ligand and cytochrome P450 2E1 (CYP450 2E1) as a receptor to establish the hepatoprotective interaction of phytoconstituents with the hepatic enzyme.

## 2 EXPERIMENTAL

### 2.1 Chemicals and reagents

Distilled water (Thomas Scientific, India), dimethyl sulfoxide (DMSO) (Fischer Scientific, India), absolute ethanol (Changshu Hongshen Fine Chemical Co. Ltd., China), Carbon tetrachloride (Loba Chemie Pvt. Ltd, India), 10% formalin (Fischer Scientific, India), diethyl ether (Fischer Scientific, India), paraffin wax (Fischer Scientific, India), hematoxylin (Alpha Chemika, Mumbai, India), xylene, eosin (Vizag Chemicals, India), standard drug the silymarin (Ponjim Huacheng Pharma, China), assay kits for liver biomarker (Accurex Biomedical Pvt. Ltd., India), and 2,2‐diphenyl‐1‐picrylhydrazyl (Wako Pure Chemical Co. Ltd, Japan) were used. All analytical‐grade chemicals and reagents were used in this study.

### 2.2 Plant material

Fresh *J. regia* bark weighing about 505 g was harvested during November and the plant was identified by a taxonomist at the Laboratory of National Herbarium and Plants, Nepal, and a voucher specimen (PUCD‐2021‐32) was preserved for future use. The collected barks were cleaned with water and were shade‐dried until absolutely dry. The dry barks about 300 g were ground into powder using an electric grinder. The moisture content of *J. regia* bark was 41.0%.

### 2.3 Experimental animals

Swiss albino rats of either sex (female for oral acute toxicity and male for the main study), 8–12 weeks old, and weighing between 250 and 300 g were used for the experiment. The rats were placed in a polypropylene cage with six rats per cage in conventional environmental conditions (25 ± 3°C and 12 h natural light–dark cycle). All animals had unlimited regular access to food and water. The animals were acclimatized in the laboratory environment for 10 days prior to the study. The study was conducted in compliance with the National Center for the Replacement, Refinement and Reduction of Animals in Research (NC3Rs) as per Animal Research: Reporting of In Vivo Experiments (ARRIVE) guidelines. All animals were fasted for 18 h before the experiment as per OECD (Organisation for Economic Co‐operation and Development) guidelines (OECD, 1996); induction of anesthesia was carried out by using mild diethyl ether and blood was collected. Anesthesia was induced by delivering 3.20% minimum alveolar concentration (MAC_50_) value for diethyl ether by using a large glass‐covered chamber (Flecknell, 2015; Miranda et al., 2011). All experimental animals were treated in accordance with the standard guidelines for the “care and use of animals in laboratories” published by the National Institutes of Health (NIH, 2010) and Institutional Review Committee approval number (Ref. No. 7‐077‐078) was acquired from Pokhara University, Nepal, prior to the commencement of in vivo research.

### 2.4 Preparation of plant extracts

Cold maceration was used to extract 300 g of bark of *J. regia* powder using absolute ethanol as the solvent. Whatman grade 1 filter paper and clean muslin cloth were used to filter the resultant extract. The liquid extract was evaporated in a rotatory evaporator (Heidolph, Germany) at 250–175 mbar pressure, 90 rpm (revolutions per minute), 40°C water bath temperature, and 5°C chilling temperature, until the solvent was completely evaporated. The concentrated filtrate was kept in a glass vial in vacuum desiccators for complete drying and further use.

### 2.5 Qualitative phytochemical analysis

Standardized tests were used to identify the presence of plant secondary metabolites in the extract (Bhatnagar et al., 2012; Vishnoi, 2000).

### 2.6 Quantitative phytochemical analysis

#### 2.6.1 Total phenolic content

Folin–Ciocalteu method was used to determine the overall phenolic content of the ethanolic extract of *J. regia* (Singleton & Rossi, 1965). In short, 5 mL of distilled water, 1 mL of the Folin–Ciocalteu reagent, and 1 mL of 1 mg/mL plant extract were combined. After 5 minutes, 1 mL of sodium carbonate (10% w/v) was added and shaken. Using a Cary 60 UV–Visible Spectrophotometer (Agilent, United States), the absorbance was observed at 725 nm after it had been left to stand for 60 min. Gallic acid (GA) (25–500 mg/L) served as the reference to quantify the overall content of the phenolic compounds, and the findings were represented as gallic acid equivalents (GAE) in milligrams for every dry weight of the extract in grams.

#### 2.6.2 Total flavonoid content

Total flavonoid content was determined according to the method explained by Zhishen et al. The flavonoid content of *J. regia* extract was quantified by colorimetric method using aluminum chloride (Zhinshen et al., 1999). Shortly, 4 mL of distilled water was mixed with 1 mL of 1 mg/mL of the plant extract. Then, 0.3 mL of 5% solution of sodium nitrite was added to the mixture followed by the addition of 0.3 mL of 20% aluminum chloride after 5 min, and the mixture was left to stand for 6 min. After that, one molar sodium hydroxide (2 mL) was added. The absorbance was measured against a blank at 510 nm by using a spectrophotometer. Quercetin was used as the standard. Per gram of dry weight of the extract's total flavonoid content was represented as milligram of quercetin equivalent.

### 2.7 Antioxidant activity

The scavenging of 2,2‐diphenyl‐1‐picrylhydrazyl (DPPH) free radicals, nitric oxide (NO) radicals, and hydrogen peroxide (H_2_O_2_) were used to measure the antioxidant activity of *J. regia* extract.

#### 2.7.1 DPPH scavenging activity

The technique mentioned by Mensor et al. was used to assess the DPPH free radicals neutralizing activity of the extract of *J. regia* (Mensor et al., 2001). Briefly, 2 mL of *J. regia* bark ethanolic extract (0.1–100 μg/mL) was combined with a solution of 2 mL DPPH (60 μM). The combination was permitted to react completely for half an hour at ambient temperature in dark. By using an ultraviolet (UV)–visible spectrophotometer, the deterioration of the purple hue of the *J. regia* extract was determined at 518 nm. As a positive control, ascorbic acid was dissolved in ethanol to create a stock solution with the same concentration. The test solution without the sample served as a negative control. The proportion of radicals scavenging capacity for DPPH was determined using the formula:

$$\%\text{radicals scavenging capacity for DPPH}=\left(C_{\mathrm{abs}}-T_{\mathrm{abs}}\right)/C_{\mathrm{abs}}\times 100$$

Whereas, *C*_abs_ and *T*_abs_ are control and *J. regia* ethanolic extract absorbance, respectively.

After plotting the inhibition percentage versus extract concentration, the antioxidant activity of the extract was determined by using a graph and represented in relation to half‐maximal inhibitory concentration (IC_50_).

#### 2.7.2 Nitric oxide radicals scavenging activity

The nitric oxide (NO) radicals scavenging test outlined by Rao was employed to determine the free radicals scavenging activity of *J. regia* (Rao, 1997). For this, 1 mL of sodium nitroprusside solution and 1 mL of the extract in a range of concentrations (0.1–100 μg/mL) were combined and kept at 29°C for 2.5 h. After incubation, the reaction mixture received 2 mL Griess reagent, and the optical density was observed using an ultraviolet (UV)–visible spectrophotometer at 548 nm. The proportion of nitric oxide radicals' inhibition by the curcumin standard and the antioxidant activity of the plant extract were estimated as follows:

$$\%\mathrm{NO}\;\text{radicals scavenging capacity}=\left(C_{\mathrm{abs}}-T_{\mathrm{abs}}\right)/C_{\mathrm{abs}}\times 100$$

Where *C*_abs_ and *T*_abs_ are control and *J. regia* ethanolic extract absorbance, respectively.

#### 2.7.3 Hydrogen peroxide (H_2_O_2_ ) scavenging activity

Using the Ruch and Co. approach, the potential of the extract to scavenge hydrogen peroxide was evaluated (Ruch et al., 1989). Shortly, 1 mL of extract (100 μg/mL) and 0.6 mL of 40 mM hydrogen peroxide made with 0.1 M of phosphate buffer (pH 7.4) were mixed. After 10 minutes of incubation, the entire volume was filled with buffer up to 3 mL and the optical density was measured at 230 nm using a ultraviolet (UV)–visible spectrophotometer using a buffer as blank. Similar to the reaction mixture, the control lacked the test sample. The following equation was used to compute the H_2_O_2_ scavenging activity:

$$\%H_{2}O_{2}\;\text{scavenging capacity}=\left(C_{\mathrm{abs}}-T_{\mathrm{abs}}\right)/C_{\mathrm{abs}}\times 100$$

where *C*_abs_ and *T*_abs_ are control and *J. regia* ethanolic extract absorbance, respectively.

### 2.8 Acute toxicity test

To investigate the oral toxicity of *J. regia* extract, OECD (Organisation for Economic Co‐operation & Development) No. 423 criteria were followed (OECD, 1996). Prior to and for 3–4 h after the extract was administered, all rats underwent overnight fasting with free access to water. For this, female nonpregnant and nulliparous healthy rats were used as per OECD guidelines. The extract was delivered orally as one dose of 2000, 4000, and 5000 mg per kg of body mass in three individual rats. The treated rats were monitored for 4 h with half‐hour intervals, and then for 2 weeks with a day's interval for the intake of food and water, death, and general toxicity manifestations, such as diarrhea, lethargy, tremor, reduced weight, and paralysis. The *J. regia* extract is considered to have a high degree of safety if no animal death was noted in a study with one rat, meaning that its fatal dose (LD_50_) is larger than 5000 mg/kg.

### 2.9 Grouping and dosing of experimental animals

Six groups of six rats (healthy male) each were used by randomly assigning the rats to the following treatments: normal control group (distilled water at 10 mL/kg), the toxic control group (distilled water at 10 mL/kg), standard test (silymarin at 100 mg/kg), and *J regia* extract at 125, 250, and 500 mg/kg each day orally for seven days, after which a single dose of 50% CCl_4_ dissolved in olive oil at a dose of (1.5 mL/kg, i.p.) was administered on the eighth day for all groups, except normal control group. The normal control group was treated with 1.5 mg/kg i.p. olive oil only. The precise amount of plant extract to be utilized for evaluating its hepatoprotective activity was determined using an oral acute toxicity test. The *J. regia* extract, silymarin, and control substances were all delivered orally through oral gavage tubes at a volume of 1 mL per 100 g. *J. regia* extract was dissolved in distilled water, which was used in both oral acute toxicity and hepatoprotective studies.

### 2.10 Hepatoprotective activity

The procedure utilized to test hepatoprotective activity was modified from that employed before by Sintayehu et al. (2012). Each rat had to undergo an 18‐hour fast before weighting. As stated earlier, the experimental animals were split up into six separate groups and dosed accordingly.

Rats were weighed the following nine days, and blood was extracted from the retro‐orbital plexus while they were lightly sedated with ether. Aspartate aminotransferase (AST), alanine aminotransferase (ALT), alkaline phosphatase (ALP), direct bilirubin (DBI), total bilirubin (TBI), total serum protein (TP), and the serum albumin levels were among the blood biochemical markers that were measured as part of the liver damage test. For the liver blood test, blood samples from each rat were collected in sterile gel tubes. These tubes were then spun in a centrifuge to release the liver biomarker, which was then analyzed by a clinical chemistry analyzer (BAS‐100 TS, Labomed Inc., USA). The rats were euthanized by using diethyl ether overdose (5% MAC_50_ value of diethyl ether in a closed glass chamber) followed by cervical decapitation (Flecknell, 2015; Miranda et al., 2011). The absolute and relative weights (organ‐to‐body weight ratio) of the liver were recorded.

The percentage of hepatoprotection of the *J. regia* extract was calculated for the evaluation of hepatoprotective activity by the following equation;

$$\%\text{Heparoprotection of}\;J.\;\text{regia}\;\text{extract}=\frac{\left(a-b\right)}{\left(a-c\right)}\times 100$$

where *a* = mean value of the hepatotoxin (CCl_4_) produced marker. *b* = mean value of hepatotoxin plus *J. regia* extract produced marker. *c* = the average value that the vehicle control produces (olive oil).

### 2.11 Histopathological analysis

The livers from all rats were removed and thoroughly washed using ice‐cold 0.9% normal saline and maintained in a sterile sample container in 10% buffered formalin at least for 24 h. Following that, repeated ethanol dehydration (70%–100%), xylene clearing, and paraffin wax embedding were performed on the liver samples. Hematoxylin and eosin were used to stain the liver tissue slices of 5 μm, which were then examined under a microscope for histological findings (Pearse, 1968). The liver histopathology images were captured at 10x original magnification.

### 2.12 Molecular docking

**FIGURE 1:**
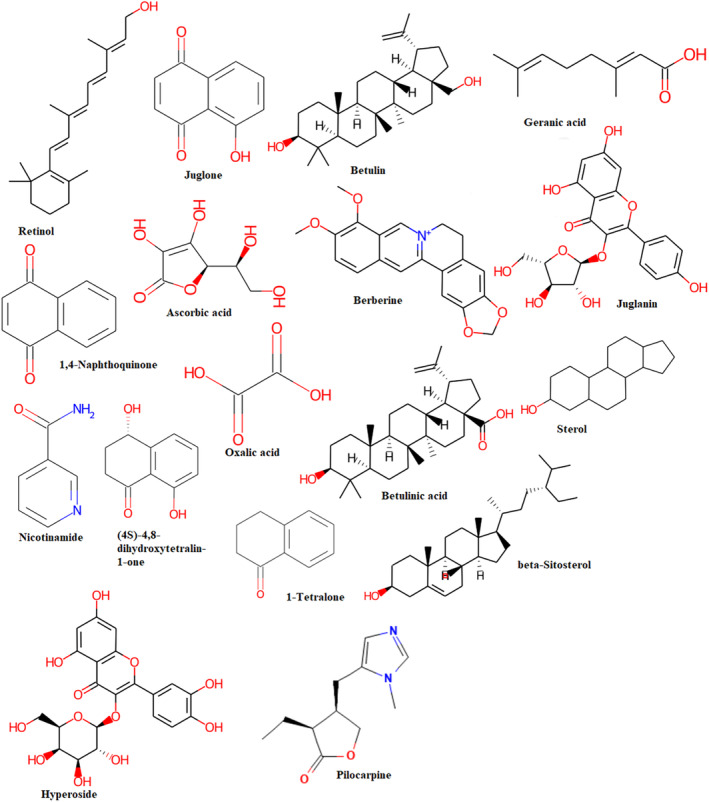
Two‐dimensional (2D) structure of selective 16 phytochemicals of *Juglans regia* bark and one co‐crystal ligand (Pilocarpine).

Various published papers confirmed the presence of variety of active constituents in *Juglans regia* bark (Jabli et al., 2017; Kale et al., 2012; Lamichhane et al., 2016). To this, we have accessed the Indian Medicinal Plants, Phytochemistry and Therapeutics 2.0 (IMPPAT 2.0) web server (https://cb.imsc.res.in/imppat/) (Vivek‐Ananth et al., 2023). From this web server, we have downloaded 16 chemical constituents (Figure 1) in three‐dimensional (3D) ‘sdf’ file format. The chirality and stereochemistry of the ligand was optimized. Merck molecular force field (MMFF94) was employed to minimize the local minima by inbuilt extension of AutoDock Vina 1.5.7 Ubuntu 20.04.6 platform. The number of torsion angles was set as default on MGLTools (Molecular Graphics Laboratory). The ‘sdf’ format of all ligands was converted into ‘pdbqt’ for molecular docking (Gote et al., 2023).

Cytochrome P450 2E1 (CYP450 2E1) enzyme (PDBID:3T3Z) of 2.3 Å X‐ray crystal structure was retrieved from the Protein Data Bank (PDB) (DeVore et al., 2012). CYP450 2E1 activates the CCl_4_ to induce the reactive species which may damage the liver. The structural coordinate was validated by the analysis of Ramachandran plot. Using the AutoDock 1.5.7 program, polar hydrogen and Kollman charge were added to the protein. One of the most important steps in molecular docking is the purification and processing of proteins. Therefore, the BIOVIA Discovery Studio Visualizer 2021 platform carefully removed complicated cofactors, water molecules, and heteroatom from the proteins (Thapa, Biradar, Banerjee, & Karati, 2023). The ‘pdb’ file of protein was converted into ‘pdbqt’ for docking by Open Babel software (O'Boyle et al., 2011).

**FIGURE 2:**
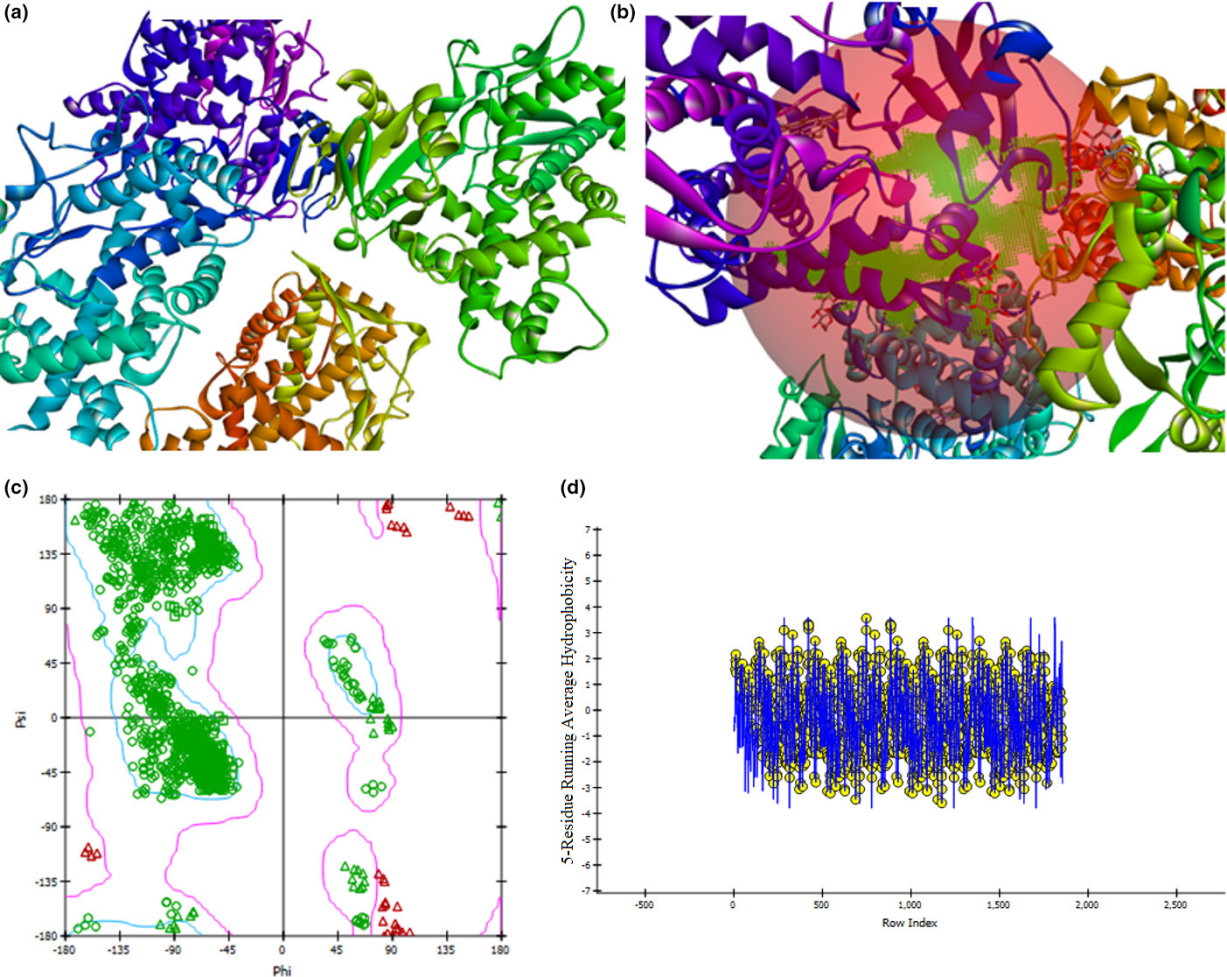
Three‐dimensional (3D) structure of CYP450 2E1 (PDBID: 3T3Z) (a), a grid box determination (b), Ramachandran plot (c), and Hydrophobic plot (d).

The Lamarckian Genetic Algorithm of Auto Dock Vina version 1.5.7 software was used to execute molecular docking on the Linux Ubuntu 20.04.6 platform (Trott & Olson, 2009). The protein without any native ligands underwent energy minimization and the amino acids were assigned as AD4 type after calculating the missing atoms (Thapa, Nargund, Biradar, Banerjee, & Karati, 2023). The grid box around the native ligand was created (Figure 2) and the dimension file was generated from the BIOVIA Discovery Studio Visualizer 2021. Then, the binding pocket was selected.

Ligands with a molecular weight above 500 Daltons were excluded from consideration for docking. The ligands and protein's “pdbqt” files were uploaded onto the system. The values of the center grid box were 50.700, −2.661, and 25.917 for X‐center, Y‐center, and Z‐center, respectively. The grid spacing and size of the box were established as 1 Å and 126 units, respectively. The value of 8 was assigned to exhaustiveness for all proteins in each docking computation. A docking command, namely the “Perl vina_linux” script, was executed to carry out the molecular docking process. The docking results were visualized using BIOVIA Discovery Visualizer 2021 version. The pose with the best docking score was selected and rendered for representation (Thapa, Nargund, & Biradar, 2023). The docking results were validated by calculating the Root Mean Square Deviation (RMSD) value by using PyMol software.

### 2.13 Retrospective searching for main components of *J. regia*

The authentic research articles were downloaded from the Scopus‐indexed journal by using “GC‐MS (Gas chromatography‐Mass spectroscopy) of *Juglans regia*” as prompt. Around eight relevant articles were selected and the main common chemical component analyzed from the articles (Al‐Rawi et al., 2021; Jabli et al., 2017; Kale et al., 2012). The ligands selected for molecular docking were validated by the GC–MS (gas chromatography–mass spectroscopy) result from previously published articles and the data stored in IMPPAT 2.0 web server (https://cb.imsc.res.in/imppat/) (Vivek‐Ananth et al., 2023) and COCONUT (Collection of Open Natural Products) (https://coconut.naturalproducts.net/) web server (Sorokina et al., 2021).

### 2.14 Statistical analysis

The findings are presented as the average (mean) and standard error of mean (SEM). SPSS version 16 software was used for statistical analysis. Analysis of variance (ANOVA) (one‐way) and the post hoc (Tukey test) test were carried out for statistical analysis. *p* values <.05 and <.001 were used to define statistically significant differences.

## 3 RESULTS AND DISCUSSION

In the present communication, phytochemical analysis, total phenolic and flavonoid content estimation, in vitro antioxidant activity, and in vivo hepatoprotective activity of ethanolic extract of the bark of *J. regia* with in silico study were evaluated. Phytochemicals are of high interest because of their substantial applications. Medicinal plants are considered as drug storehouses for traditional medicine systems, allopathic medicine systems, folk medicine, food supplements, intermediate products of pharmaceuticals, nutraceuticals, and chemical entities for various synthetic modern medicines (Ncube et al., 2008).

The liver has a requisite function in life due to its role in metabolic and detoxification actions (Muriel & Rivera‐Espinoza, 2008). When the liver is subjected to various endogenous and xenobiotic agents, numerous intermediate and finished compounds are produced. These agents are the principal causes of hepatocellular necrosis and finally disease of the liver (Lee, 2003; Wei et al., 2024). To confirm the protection of hepatocellular damage and maintain a healthy liver, the current existing treatment attends to symptomatic treatment and hepatic transplant in severe hepatic disease (Lampertico et al., 2017). Yet, there is no other better therapeutic candidate for increasing the detoxification capacity of the liver. In this regard, testing and utilization of natural‐based hepatocurative agents are significantly soaring. Since it would be very crucial to exhibit how well the plant extracts function to cut off the liver injury caused by xenobiotics. CCl_4_ is a commonly used hepatotoxic substance in order to assess the ability of the plant extract to protect the liver (Arshad et al., 2024; Pritchard & Apte, 2015). The human scenario is closely simulated by in vivo experimental rodent models, which also make it simple to measure biochemical and histopathological characteristics (Arshad et al., 2024; De et al., 2017). In order to evaluate the in vivo liver‐protective efficacy of the ethanolic extract of the bark of *J. regia*, the CCl_4_‐induced hepatotoxicity in rats was used as the experimental model in this investigation. Both humans and laboratory animals can develop centrizonal hemorrhagic hepatic necrosis as a result of CCl_4_ (De et al., 2017). In this research, the silymarin was used as the standard hepatoprotective agent, which is also used as a standard positive control in most extensively researched plant extracts in preventing the liver injury experimental models (Dawada et al., 2012).

### 3.1 In vitro study

#### 3.1.1 Extraction yield, nature of the extract, and phytochemical screening

The extraction yield percentage of the ethanolic extract of the barks of *J. regia* was 9.87% with dark chocolate‐colored solid nature extract. Qualitative screening of phytochemicals from the extract of *J. regia* indicated the occurrence of alkaloids, carbohydrates, phenolics, flavonoids, saponins, and terpenoids. However, glycosides and amino acids were absent in the extract.

#### 3.1.2 Overall content of phenolics and flavonoids

**FIGURE 3:**
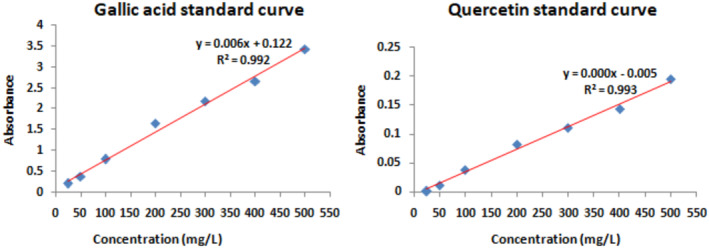
Gallic acid and quercetin standard calibration curve for calculation of total phenolic and falvonoid contents.

Calibration curve of gallic acid and quercetin which were taken as standard at seven different concentrations 25 to 500 mg/L was drawn, as shown in Figure 3. On the basis of the standard regression line for gallic acid (*y* = 0.006*x* + 0.122; *R* ^2^ = .992) and quercetin (*y* = 0.000*x* − 0.005; *R* ^2^ = .993), the equivalent total phenolic content and total flavonoid content were calculated. *J. regia* extract showed 496.67 ± 1.02 milligram GE/g dry extract weight and 2028.02 ± 0.19 milligram QE/g dry extract weight of phenol and flavonoid content, respectively (Figure 3).

In accordance with prior research, *J. regia* ethanolic extract includes active phytoconstituents such as polyphenols, alkaloids, flavonoids, terpenoids, carbohydrates, and amino acids that may have hepatoprotective properties alone or in combination (Doss, 2009). The ethanolic extract of the barks of *J. regia* displayed significant levels of total phenolic and flavonoid contents. These results are in line with the results of past studies that showed the hepatoprotective action of these phytochemicals (Alghazeer et al., 2018).

#### 3.1.3 Antioxidant activity

Ethanolic extract of the bark of *J. regia* showed the highest DPPH free radicals scavenging activity, i.e. 94.71 ± 0.33 percentage of radicals scavenged with IC_50_ value (2.31 μg/mL) at the concentration of 100 μg/mL compared to ascorbic acid. In contrast, moderate and lower percentage of radicals scavenging capacity was found for the scavenging of NO and H_2_O_2_ radicals, respectively (Table 1).

**TABLE 1:** Percentage of DPPH, NO, and H_2_O_2_ radical acts of scavenging of *J. regia* extract at various concentrations.

S. No.	Antioxidant assay method	Test sample and standard	Test concentration				IC_50_ value (μg/mL)
			0.1 μg/mL	1 μg/mL	10 μg/mL	100 μg/mL	
1.	DPPH free radical scavenging	*J. regia* extract	58.20 ± 2.59	67.86 ± 0.73	88.97 ± 4.72	94.71 ± 0.33	2.31
		Standard (ascorbic acid)	56.83 ± 2.86	63.15 ± 5.4	95.16 ± 1.97	97.83 ± 0.06	2.44
2.	NO free radical scavenging	*J. regia* extract	26.98 ± 7.13	29.78 ± 6.97	37.28 ± 3.59	48.85 ± 1.45	35.66
		Standard (curcumin)	27.09 ± 4.11	29.59 ± 4.64	33.35 ± 3.45	42.22 ± 1.93	79.60
3.	H_2_O_2_ radical scavenging	*J. regia* extract	–	–	–	28.95 ± 4.51	–
		Standard (ascorbic acid)	–	–	–	87.99 ± 0.2	–

*Note*: Average ± SEM (standard error of mean) (*n* = 3) is used to express all data.

A single antioxidant assay method is not sufficient for the assessment of the antioxidant potential of plant extracts. Since different harmful radicals scavenging assays use different experimental techniques and have different assay principles. For instance, this study employed the DPPH free radical assay, NO radicals scavenging assay, and the hydrogen peroxide assay technique. Both phenol and flavonoid compounds show medicinal activity such as antibacterial, antiviral, anti‐inflammatory, anticancer, antiallergic, and hepatoprotective activities, making them capable of scavenging singlet oxygen and other free radicals (Bravo, 1998; Montoro et al., 2005). Our findings, which were in line with the earlier discovery, revealed that high phenolic and flavonoid contents are associated with high levels of harmful radicals scavenging potential when assessed by adopting the DPPH free radicals test technique (Sahreen et al., 2011). However, no association was found in the case of scavenging hydrogen peroxide radicals. This may be because, depending on the chemical makeup, the antioxidant activity of the phenolic compounds varies greatly (Satué‐Gracia et al., 1997). In addition, some chemical constituents, such as sugar or ascorbic acid present in the extract, also interfere with the radicals scavenging activity (Singleton & Rossi, 1965).

As reported by Trifunschi et al., plant extracts showed hepatoprotective activity mainly because of their capacity to scavenge free radicals (Trifunschi et al., 2015). As CCl_4_‐induced toxicity generates inflammatory mediators such as monocytes, neutrophils, interleukin‐6 (IL‐6), and tumor necrosis factor‐alpha, plants exhibit anti‐inflammatory action for hepatoprotection (Yoshioka et al., 2017). The decrease in the serum liver indicators further suggests that flavonoids may protect against CCl_4_‐induced cell membrane instability and damage. It is in agreement with the outcome of the study conducted by Tarahovsky et al. (2014). Alkaloids, saponins, and flavonoids, among other plant secondary metabolites, have hepatoprotective effects by acting as anti‐inflammatory agents. This result is consistent with prior research that has demonstrated the anti‐inflammatory properties of phytoconstituents (Huang et al., 2017).

#### 3.1.4 Molecular docking

In this molecular docking study, 16 phytochemicals were evaluated against the CYP450 2E1 enzyme using computational methods. Molecular docking allows us to predict the binding affinity and interactions between small molecules (the phytochemicals) and the target protein (CYP450 2E1) to understand their potential as inhibitors. Cytochrome P450 2E1 enzyme (PDBID:3T3Z) contains a native ligand which is Pilocarpine. As regards the structure, the Pilocarpine is a heterocyclic imidazole derivative. This substance is derived from plants belonging to the genus *Pilocarpus*. *Pilocarpus microphyllus* is the exclusive source of Pilocarpine, and its commercial manufacture is solely obtained from the leaves of this plant (Hancock, 2007). Since the native ligand is a plant‐based imidazole derivative, it has some structural similarity to 16 ligands selected by us. Therefore, 3T3Z was chosen as protein/target for the docking study and the results were compared with those of the native ligand Pilocarpine.

**FIGURE 4:**
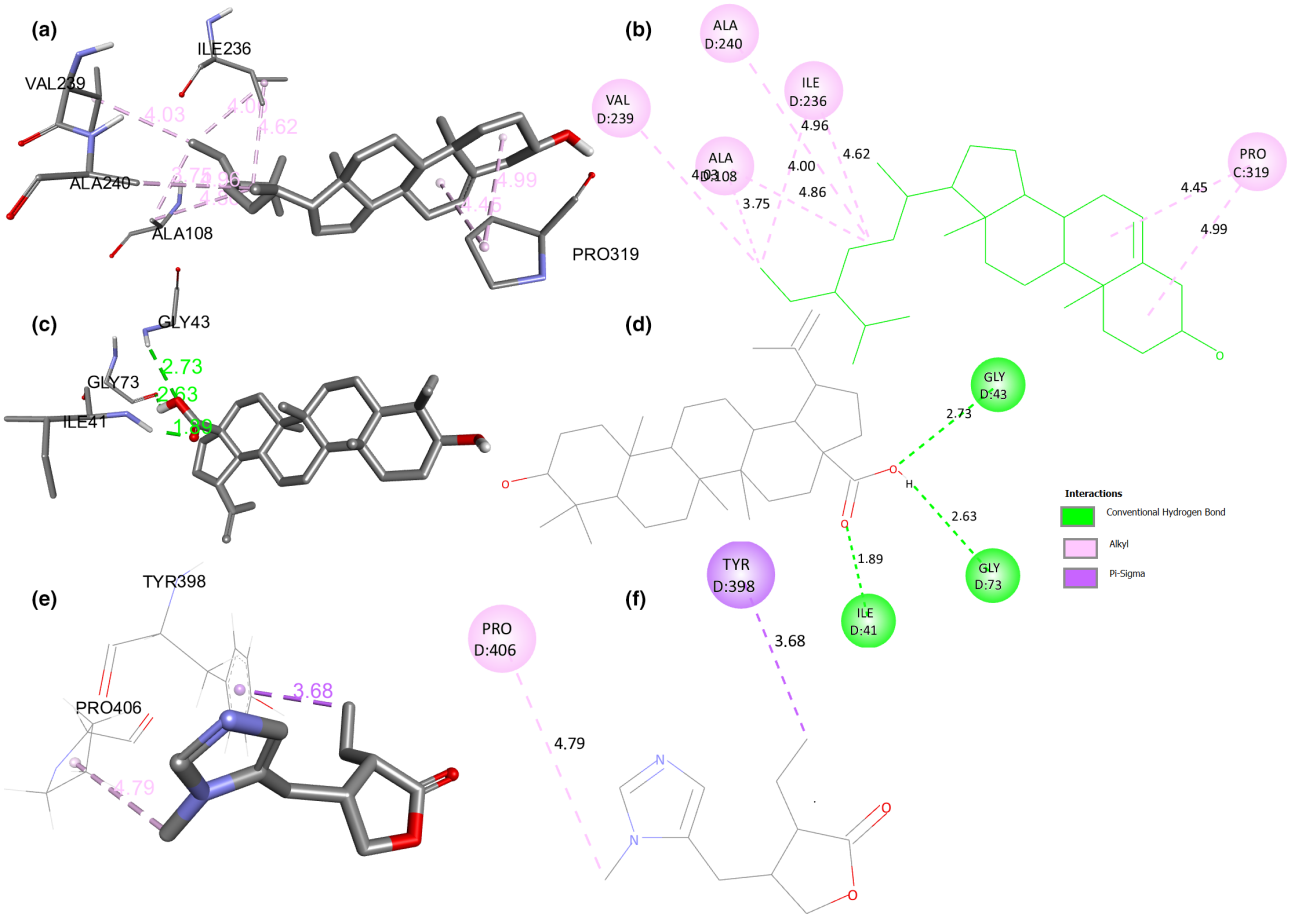
Two‐dimensional and three‐dimensional (2D and 3D) molecular docking interaction of Beta‐Sitosterol (a, b), Betulinic acid (c, d), and Pilocarpine (e, f) with CYP450 2E1 (PDBID: 3T3Z).

Leu197, Leu171, Ala157, Pro319, Ile236, Ala108, Val108, Ala240, Leu45, and Phe46 were the most common amino acids that participated in the interaction (Supplementary Materials, Figures S1). The binding energies observed ranged from −4.3 to −9.2 kcal/mol (Table 4), indicating varying degrees of affinity between the phytochemicals and the enzyme. Lower binding energies suggest stronger binding interactions between the compounds and the enzyme. Two compounds that were particularly noteworthy were Betulinic acid and Beta‐sitosterol, which showed significant binding energies of −9.1 and −9.2 kcal/mol, respectively (Figure 4). Furthermore, Betulin and Sterol also showed significant binding energies of −8.9 and −8.2 kcal/mol. Interestingly, 1‐tetralone and hyperoside exhibited a similar binding energy of −8.0 kcal/mol. These values suggest a strong affinity of these compounds for the active site of the CYP450 2E1 enzyme, potentially indicating their efficacy as inhibitors. All the 16 ligands showed RMSD value less than 2 Å (Table 4).

In comparison to the native ligand Pilocarpine, which had a binding energy of −5.5 kcal/mol, both Beta‐sitosterol and Betulinic acid had considerably stronger binding affinity. This suggests that they have the potential to be more effective compounds in their interaction with CYP450 2E1. Additionally, all the phytochemicals showed the stronger affinity than the co‐crystal native ligand (Pilocarpine) against the enzyme, except oxalic acid (−4.3 kcal/mol). The precise interactions observed during the molecular docking approach offer additional understanding of the binding mechanisms of these drugs. Beta‐sitosterol exhibited six pi‐alkyl interactions, indicating that its binding to the enzyme involves hydrophobic interactions. Conversely, Betulinic acid displayed three typical hydrogen bonds, suggesting a distinct mechanism of interaction characterized by specific hydrogen bonding with the active site residues of the enzyme. Because Betulinic acid exhibits hydrogen bonding, it may be a superior phytoconstituent for future research to consider as a potential therapeutic candidate, as hydrogen bonding is crucial to the molecular docking process that establishes the ligand–receptor‐binding mechanism (Chen et al., 2016). CYP450 2E1, a hepatic enzyme, metabolizes the hepatotoxic agent carbon tetrachloride (CCl_4_) in rat models. CYP450 2E1 activates CCl_4_, generating highly reactive radicals that induce oxidative stress, leading to lipid peroxidation, protein, and DNA damage in hepatocytes. Elevated CYP450 2E1 expression intensifies reactive oxygen species (ROS) production, exacerbating oxidative stress in the liver. Consequently, cellular damage, inflammation, and hepatocyte necrosis occur. Modulating CYP450 2E1 activity is explored as a potential strategy to mitigate CCl_4_‐induced liver damage (Khan et al., 2012; Shaban et al., 2023; Xu et al., 2017). The dried bark of *J. regia* contains bioactive polyphenols (NirmlaDevi et al., 2011). However, a number of compounds, including Beta‐sitosterol, ascorbic acid, juglone, folic acid, gallic acid, regiolone, Betulinic acid, and quercetin‐3‐α‐L‐arabinoside, show hepatoprotective activity, which have been isolated from the *J. regia* bark (Zakavi et al., 2013). A similar result was expressed by our in silico study. Beta‐sitosterol (−9.2 kcal/mol) and Betulinic acid (−9.1 kcal/mol) return the strong affinity toward the CYP450 2E1 enzyme. It is noteworthy to mention that both the complexes have strong stability, which was determined by the RMSD value (less than 2 Å).

We found some interesting information about the binding modalities in the interactions between Betulinic acid and the CYP450 2E1 enzyme. The binding profile of Betulinic acid was highly significant, since it formed three hydrogen bonds with essential amino acid residues located in the active region of the enzyme. More precisely, these interactions took place with Gly43 at a distance of 2.73 Å, Gly73 at a distance of 2.63 Å, and Ile41 at a very close distance of 1.89 Å (Figure 4). The carboxylic acid group (‐COOH) of Betulinic acid plays a crucial role in creating these hydrogen bonds, as seen by its involvement in all three interactions. The results of this study clarify the specific mechanism by which Betulinic acid binds to CYP450 2E1 and demonstrate its capacity to block the activity of enzyme. These findings provide an important information for future experimental investigations and the development of pharmaceuticals that target CYP450 2E1.

These results emphasize the potential of Betulinic acid and Beta‐sitosterol as interesting candidates for further experimental validation and possible development as inhibitors of the CYP450 2E1 enzyme. Beta‐sitosterol has the potential to be an excellent choice for development as a therapeutic candidate due to its ability to inhibit through hydrophobic interactions. On the other hand, Betulinic acid inhibits through conventional hydrogen interactions. This is owing to their substantial binding affinities and unique ways of interacting with the target enzyme.

#### 3.1.5 Main components of *J. regia* reported from database and articles

To validate the presence of different phytoconstituents, we have investigated the published articles and different plant databases like COCONUT and IMPPAT 2.0. We observed different secondary metabolites recorded in the database, which we then verified by comparing with published spectral data. We only mention the reported phytoconstituents having GC–MS data in Table 5 (some fatty acids or long‐chain hydrocarbons, which are not pharmaceutically important, are excluded from the list of ligands). Different spectral studies revealed the presence of variety of aromatic and heterocyclic components in *J. regia* bark, which may help in the hepatoprotective activity. The prominent components of *J. regia* bark include sterol, steric acid, oleic acid, n‐heptadecanoic acid, palmitic acid, n‐octadecane, etc (Table 5).

### 3.2 In vivo study

#### 3.2.1 Oral acute toxicity study

Oral administration of the ethanol extract of *J. regia* bark did not exhibit any alterations in eating amount, and behavioral changes including changes in movement, activity, hair texture, and pupil size. At a dosage of 5000 mg/kg, no morbidity or mortality was seen. The fatal dose (LD_50_) of the *J. regia* extract is categorized as Globally Harmonized System (GHS) Category 5 since its estimated concentration is more than 5000 mg/kg, which is confirmed and tested by/as per the OECD oral acute toxicity test. Since *J. regia* extract up to 5000 mg/kg is safe for oral administration to rat.

#### 3.2.2 Body and liver weight of the experimental animals

Table 2 lists the starting and ending body weight, liver weight, and the relative liver weight of the six experimental groups that were sacrificed after the first eight days of the research. Over the 8‐day study period of the experiment, there were no discernible changes observed in the general conditions of the animals in either group. Comparing the CCl_4_‐treated group with the usual control group, there was a reduction in body weight. In contrast to the normal control, the toxic control, and standard test groups, the rats treated with ethanolic extract of *J. regia* bark at dosages of 250 and 500 mg/kg revealed significantly lower liver weight (*p* < .05).

**TABLE 2:** Experimental animals’ body weight, liver weight, and weight gain.

Parameters	Experimental group					
	Normal control group (DW; 10 mL/kg)	Toxic control group (DW; 10 mL/kg)	Standard group (Silymarin; 100 mg/kg)	*J. regia* extract (500 mg/kg)	*J. regia* extract (250 mg/kg)	*J. regia* extract (125 mg/kg)
Initial body weight (g)	276.50 ± 22.39	241.84 ± 13.80	231.84 ± 6.64	285.67 ± 28.65	260.50 ± 21.39	258.34 ± 19.74
Final body weight (g)	300.16 ± 22.38	260.67 ± 17.11	250.16 ± 10.31	290.84 ± 29.27	265.67 ± 23.87	270.50 ± 19.87
Weight gain (g)	23.67 ± 1.20	18.84 ± 2.54	18.84 ± 4.10	5.16 ± 1.97^a–c^	5.16 ± 3.26^a–c^	12.16 ± 3.74
Liver weight (g)	10.62 ± 1.12	11.40 ± 0.78	10.84 ± 0.93	12.79 ± 1.36	10.20 ± 1.33	11.51 ± 1.52
Relative liver weight	3.49 ± 0.14	4.44 ± 0.38	4.31 ± 0.25	4.55 ± 0.64	3.79 ± 0.23	4.18 ± 0.38

*Note*: The results depict the average and standard error of mean (SEM) of six rats. Administered vehicle: DW (distilled water), olive oil, and CCl_4_ in 1:1 ratio at a dose of 1.5 mL/kg was administered via i.p. route on eighth day with all groups, except the normal control group. Relative liver weight = (Liver weight/Body weight) × 100. ^a–c^ *p* < .05 significantly different from the normal, toxic, and standard control group, respectively.

**FIGURE 5:**
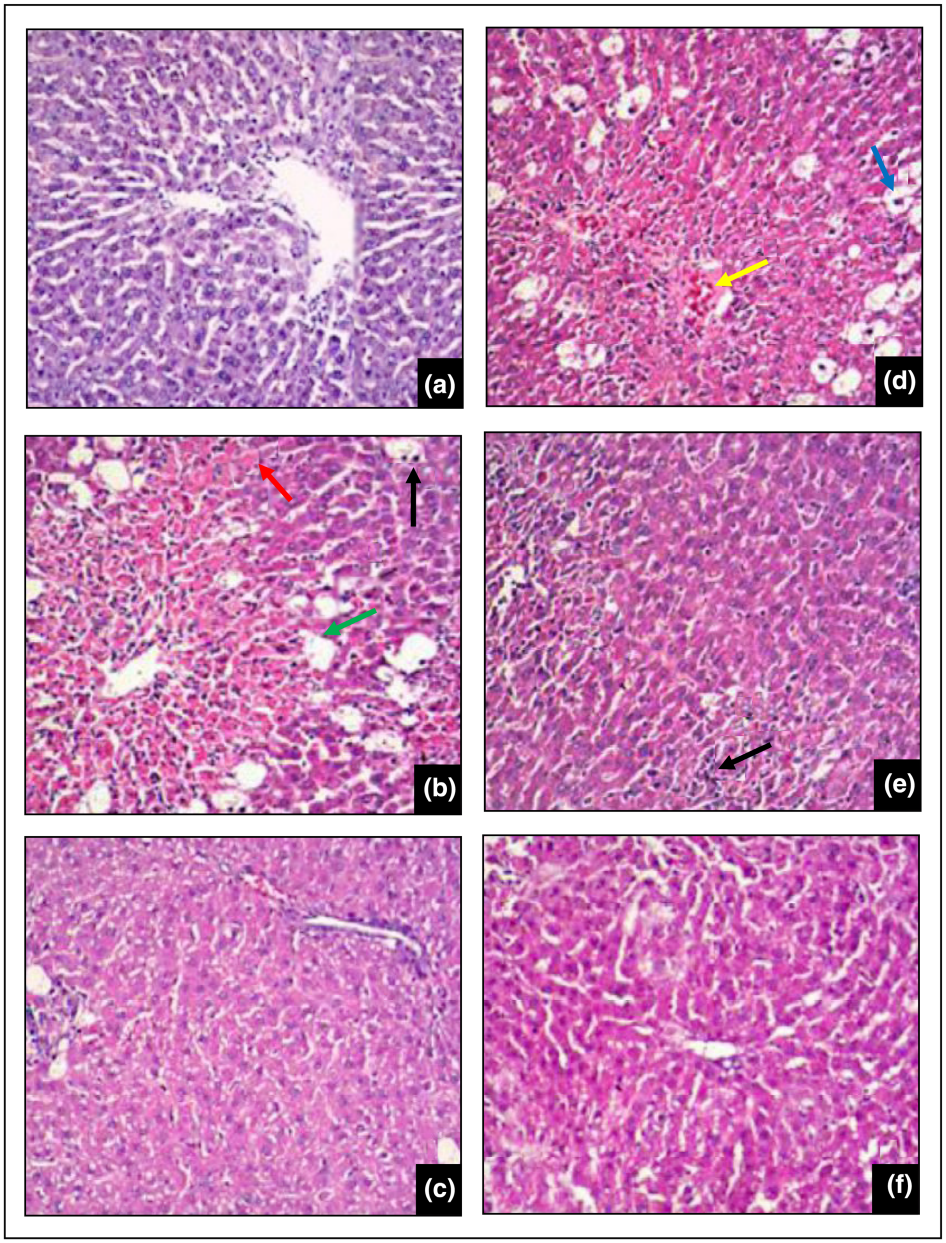
Effect of *J. regia* bark ethanolic extract on rats’ liver histology following CCl_4_‐induced hepatic damage. (a) Normal group; (b) Toxic group (CCl_4_ only); (c) CCl_4_ + 100 mg per kg silymarin; (d) CCl_4_ + 125 mg per kg *J. regia* extract; (e) CCl_4_ + 250 mg per kg *J. regia* extract; and (f) CCl_4_ + 500 mg per kg *J. regia* extract. (×100), *n* = 6. (green arrow line): degeneration of ballooning significantly; (blue arrow line): fatty alterations; (red arrow line): necrosis of hepatic cells; (black arrow line): infiltration of inflammatory cells; (yellow arrow line): conspicuous congestion.

Rats treated with only CCl_4_ showed increase in the liver weight due to the emergence to the liver infiltration, vacuolization, and inflammation (Table 2, Figure 5). Gain to the liver weight is a sign of hepatic damage and may be brought on by the retention of water in the cytoplasm of hepatocytes, which causes the liver cells to expand and ultimately result in an increase in the total liver weight (Mahmood et al., 2014; Mulla et al., 2009).

#### 3.2.3 Impact of the ethanolic extract of the bark of *J. regia* on the serum level of liver biomarker

**FIGURE 6:**
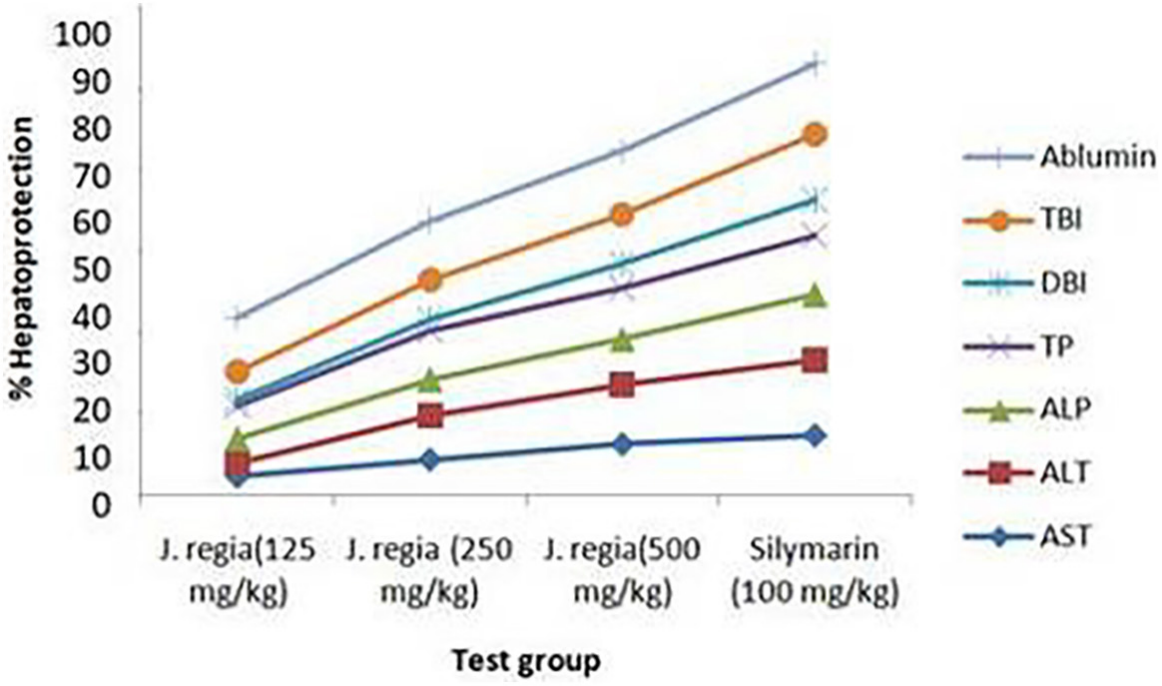
Percentage of hepatoprotection of *J. regia* extract at different doses.

The results of oral administration of extracts from *J. regia* bark on the different liver biomarker levels in the serum of rats with the injured liver are depicted in Table 3 and Figure 6. The extract of *J. regia* at 250 and 500 mg/kg diminished the serum levels of ALT, ALP, DBI, and TBI of rats with hepatic damage highly significantly (*p* < .001). Similarly, *J. regia* extract (250 and 500 mg/kg) increased the serum albumin levels of rats with significant liver injury (*p* < .001) compared to the toxic control group. The extract of *J. regia* 125 mg per kg also lowered the serum levels of ALT, ALP, TBI and increased the serum albumin levels of rats with the liver injury with significant variation (*p* < .005). Dosage of 100 mg per kg silymarin as the standard also had highly significant (*p* < .001) differences from the toxic control group for reducing the serum ALT, ALP, direct bilirubin (DBI), and total bilirubin (TBI) levels and increasing the serum albumin levels.

**TABLE 3:** Effect of the *J. regia* extract on serum biochemical parameters for the liver injury tests.

Parameters	Experimental group					
	Normal control (10 mL/kg DW)	Toxic control (10 mL/kg DW)	Standard test (Silymarin 100 mg/kg)	*J. regia* extract (500 mg/kg)	*J.regia* extract (250 mg/kg)	*J. regia* extract (125 mg/kg)
AST	131.02 ± 2.75	372.45 ± 3.74	196.72 ± 2.09 (72.78)	223.52 ± 3.48 (61.68)	269.96 ± 3.51 (42.45)	320.75 ± 5.31 (21.41)
ALT	27.4 ± 1.13	115.87 ± 9.25^b^	33.58 ± 1.13^d^ (93.01)	51.19 ± 0.94^d^ (73.1)	66.99 ± 1.71^d^ (55.25)	93.99 ± 1.07^c^ (17.94)
ALP	134.01 ± 1.48	344.98 ± 15.90^b^	176.79 ± 2.88^d^ (79.72)	226.90 ± 2.66^d^ (55.97)	251.73 ± 1.70^d^ (44.2)	281.15 ± 0.93^d^ (30.25)
TP	8.51 ± 0.07	7.25 ± 0.07	8.18 ± 0.01 (73.8)	8.06 ± 0.02 (64.28)	8.01 ± 0.01 (60.31)	7.76 ± 0.04 (40.47)
DBI	0.21 ± 0.02	0.96 ± 0.003^b^	0.63 ± 0.01^d^ (44)	0.74 ± 0.01^d^ (29.34)	0.86 ± 0.001^d^ (13.34)	0.91 ± 0.08 (6.67)
TBI	0.38 ± 0.007	0.80 ± 0.03^b^	0.46 ± 0.003^d^ (80.95)	0.54 ± 0.01^d^ (61.9)	0.59 ± 0.01^d^ (50)	0.65 ± 0.01^d^ (35.71)
Albumin	3.84 ± 0.14	0.46 ± 0.003^a^	3.42 ± 0.20^d^ (87.57)	3.14 ± 0.07^d^ (79.28)	2.89 ± 0.02^d^ (71.89)	2.69 ± 0.03^c^ (65.97)

*Note*: The results represent the mean and standard error of mean (SEM) of six rats. Numbers in parentheses indicate % hepatoprotection.

Abbreviation: DW, distilled water.

^a^ *p* < .05 substantially different from the normal group (given DW only).

^b^ *p* < .001 considerable variation with the normal group (given DW only).

^c^ *p* < .05 substantially different from the toxic group (group was given DW and CCl_4_).

^d^ *p* < .001 considerably different from the toxic group (group was given DW and CCl_4_).

**TABLE 4:** Binding energy, amino acid involved in interaction, and RMSD value of 16 ligand molecules.

SN	Name of compounds	Binding energy (kcal/mol)	Amino acids	RMSD value (Å)
1.	Retinol	−7.2	Leu197, Leu171, Ala157	1.388
2.	Juglone	−6.0	Tyr349, Lys324	0.996
3.	Betulin	−8.9	Ala240, Pro466	0.926
4.	Geranic acid	−6.3	Leu48, Glu49, Leu50, Phe B:46, Phe D:46, Leu D:45, Leu B:45, Ile42	1.359
5.	Juglanin	−7.8	Lys461, His226, Pro462, Leu460, Pro319, Ala105, Ala108	1.004
6.	Berberine	−7.2	Lys55, Pro54, Phe360	1.038
7.	Ascorbic acid	−5.6	Arg233, His226	0.584
8.	1,4‐Naphthoquinone	−5.8	Ile469, Val464	1.030
9.	Nicotinamide	−5.8	Leu48, Leu B:45, Leu B:46, Leu D:45, Leu D:46	1.013
10.	Oxalic acid	−4.3	Arg126, Arg435, Arg100	0.379
11.	(4S)‐4,8‐dihydroxy‐tetralin‐1‐one	−7.1	Leu45, Leu48, PheD: 46, PheB:46	1.072
12.	Betulinic acid	−9.1	Gly43, Gly63, Ile41	1.706
13.	Sterol	−8.2	Leu45, PheB:46, PheD:46	1.045
14.	1‐Tetralone	−8.0	Leu45, Phe46	0.871
15.	Beta‐sitosterol	−9.2	Pro319, Ile236, Ala108, Val108, Ala240	1.108
16.	Hyperoside	−8.0	Pro462, Pro319, Ile236, Ala108, Pro104	1.080
17.	Pilocarpine	−5.5	Try398, Pro408	–

**TABLE 5:** Gas chromatography–mass spectroscopy (GC–MS) profiling of active component of *J. regia* (Al‐Rawi et al., 2021; Kale et al., 2012).

SN	Main component of *J. regia*
1.	Campesterol
2.	Stigmasterol
3.	Citric acid
4.	17‐Octadecynoic acid
5.	Linoleic acid ethyl ester
6.	9,12‐Octadecadienoic acid
7.	13‐Octadecenoic acid
8.	Stearic acid
9.	Lactic acid
10.	Ethanimidic acid
11.	Oleic acid
12.	Palmitic acid
13.	n‐Heptadecanoic acid
14.	n‐Octadecane
15.	Beta‐sitosterol
16.	Betulinic acid

The liver biomarker levels in the serum, such as ALT, ALP, DBI, and TBI, were substantially different (*p* < .001) in the toxic control group in contrast with the normal group. In contrast to the normal group, the albumin levels in the toxic group were substantially different (*p* < .05). *J. regia* extract showed high percentage of hepatoprotection against CCl_4_‐induced elevated levels of AST, ALT, ALP, TBI, and DBI, and decreased TP and serum albumin level in liver as a dose‐dependent manner (high at 500 mg/kg), as shown in both Table 3 and Figure 6.

Carbon tetrachloride (CCl_4_)‐induced hepatotoxicity elevates the levels to the liver enzymes, namely, total bilirubin, alanine aminotransferase, aspartate aminotransferase, and alkaline phosphatase, which are released in the blood during liver injury. The disruption of polyribosomes on the endoplasmic reticulum, which lowers protein production, also results in decreased levels of total protein and the serum albumin (Alqasoumi & Abdel‐Kader, 2012). In our research, treatment with the *J. regia* bark extract at doses (500, 250, and 125 mg/kg) resulted in a significant reduction of the serum enzymes such as ALT, ALP, DBI, and TBI induced by the CCl_4_. Despite this, *J. regia* bark extract also helped to increase the serum albumin and protein levels caused by CCl_4_.

The liver blood test of rats treated with CCl4 showed that the treatment with ethanolic extract of *J. regia* bark suppressed the serum ALT and ALP enzyme levels, validating the plasma membrane stability and healing of the CCl4‐induced hepatocyte damage. This is consistent with earlier research conducted on other plant extracts (Gutiérrez & Solís, 2009; Zahira et al., 2023). Similarly, the decrease in the serum levels of DBI and TBI by *J. regia* bark extract in the CCl_4_‐treated group is an indication of stabilization of biliary dysfunction and improvement of metabolic function of the liver. The extract reversed the unfavorable effects of CCl_4_ on total protein and the serum albumin, indicating an increase in the functional state of the hepatic cell to generate proteins. These results were concurring with the previous studies (Shrivastava & Bhambar, 2017).

#### 3.2.4 Histopathological studies

Figure 5 depicts the histological characteristics of the rat liver from the normal control, the toxic control, positive control, and experimental groups. Histological observation of Figure 5d–f further supports the ability of the extract of *J. regia* to restore the cellular integrity in a dose‐dependent manner, hence confirming its hepatoprotective effect. With significant fatty alterations, ballooning degeneration, necrosis, and inflammatory cell attack, the toxic control group (CCl_4_ alone) demonstrated a clear breakdown of the hepatic cell architecture. The dose of 125 mg/kg of the extract with CCl_4_ provided less hepatoprotection with fatty alterations and noticeable congestion. However, the dose of 250 mg/kg of the extract with CCl_4_ showed less liver damage with noticeable hepatic inflammation. Administration of *J. regia* ethanolic extract at a dose of 500 mg/kg showed a marked curative effect in damaged hepatic cell architecture by CCl_4_.

The liver histology from normal control‐treated groups showed a normal hepatic lobule, hepatocyte cell structure, and hepatic architecture, while the toxic control‐treated group exhibited severe damage to the liver section, cellular necrosis, fatty changes, ballooning degeneration, fibrosis, and the liver inflammation in some areas. This finding was concurring with that of a previous study (Fahmy, 2016). The damage of hepatic cell architecture induced by CCl_4_ was prevented by *J. regia* extract in a dose‐dependent manner, which is comparable to that of the silymarin‐treated group.

## 4 CONCLUSION

The extract from *J. regia* bark exhibited strong antioxidant activity by effectively scavenging DPPH radicals, which can be attributed to its high levels of flavonoids and phenolic content. The hepatoprotective effects of *J. regia* extracts were demonstrated by a significant reduction in ALT, ALP, DBI, and TBI levels, as well as a significant increase in serum albumin levels compared to the toxic control group. These findings were further supported by the liver histopathological study. Our study provides compelling evidence that administering a 500 mg/kg dose of ethanolic extract from the bark of *J. regia* can effectively reduce liver damage in rats with CCl_4_‐induced liver toxicity. This shows that the extract has potential as a hepatoprotective agent. Furthermore, the results of molecular docking support the hepatoprotective effect of *J. regia* by inhibiting the CYP450 2E1 enzyme. This inhibition is attributed to the high affinity of Beta‐sitosterol (−9.2 kcal/mol) and Betulinic acid (−9.1 kcal/mol) against CYP450 2E1. The study suggests that conducting a preclinical investigation on *J. regia* is strongly advised in order to produce a powerful hepatoprotective drug from this plant. Subsequent studies can evaluate the influence of preventive measures on other organs.

## AUTHOR CONTRIBUTIONS

**Bipindra Pandey:** Conceptualization (lead); data curation (lead); formal analysis (lead); investigation (lead); methodology (lead); resources (lead); software (equal); visualization (lead); writing – original draft (lead). **Shankar Thapa:** Formal analysis (equal); software (equal); visualization (equal). **Atisammodavardhana Kaundinnyayana:** Investigation (equal); resources (equal); supervision (supporting); validation (equal); writing – review and editing (equal). **Sushil Panta:** Investigation (lead); methodology (equal); project administration (lead); resources (lead); supervision (lead); validation (equal); writing – review and editing (supporting).

## FUNDING INFORMATION

This research did not receive any specific grant from funding agencies in the public, commercial, or not‐for‐profit sectors.

## CONFLICT OF INTEREST STATEMENT

The authors have no conflicts of interest to declare for this study.

## ETHICAL CONSIDERATION

All experimental animals were handled according to the widely accepted Guide for the Care and Use of Laboratory Animals for handling and using animals released by the National Institutes of Health (NIH, 2011). Prior to starting the animal experiments, Institutional Review Committee approval (Ref. No. 7‐077‐078) was obtained from Pokhara University, Pokhara, Nepal.

## PRESENTATION AT A MEETING

Presented at Digital NAFLD Summit 2021 organized by the European Association for the Study of the Liver (EASL), Home of Hepatology, Official registration agency Congrex, Switzerland, September, 2021.

## Supporting information


Data S1.

## Data Availability

All data and materials for this study will be provided upon request to the corresponding author. Supplementary file link.
