# Supplementary material for: Hepatoprotective effects of Juglans regia on carbon tetrachloride‐induced hepatotoxicity: In silico/in vivo approach
Source: Food Sci Nutr. 2024 Jun 18;12(9):6482–97. doi: 10.1002/fsn3.4288 (PMC11561823; doi:10.1002/fsn3.4288)
Supplement: Supplementary file 1 — Data S1. [file FSN3-12-6482-s001.docx]

**Hepatoprotective effects of *Juglans regia* on carbon tetrachloride induced hepatotoxicity: In-silico/In-vivo Approach**

Bipindra Pandey^a, b*^,Shankar Thapa^a^, Atisammodavardhana Kaundinnyayana^b^, Sushil Panta^b^

^a^Department of Pharmacy, Madan Bhandari Academy of Health Sciences, Hetauda, Nepal

^b^School of Health and Allied Sciences, Pokhara University, Pokhara, Nepal

***Corresponding author:** Lecturer, Department of Pharmacy, Madan Bhandari Academy of Health Sciences, Nepal. E-mail address: [bipindra.pandey@mbahs.edu.np](mailto:bipindra.pandey@mbahs.edu.np) (Bipindra Pandey)

**Supplementary materials**


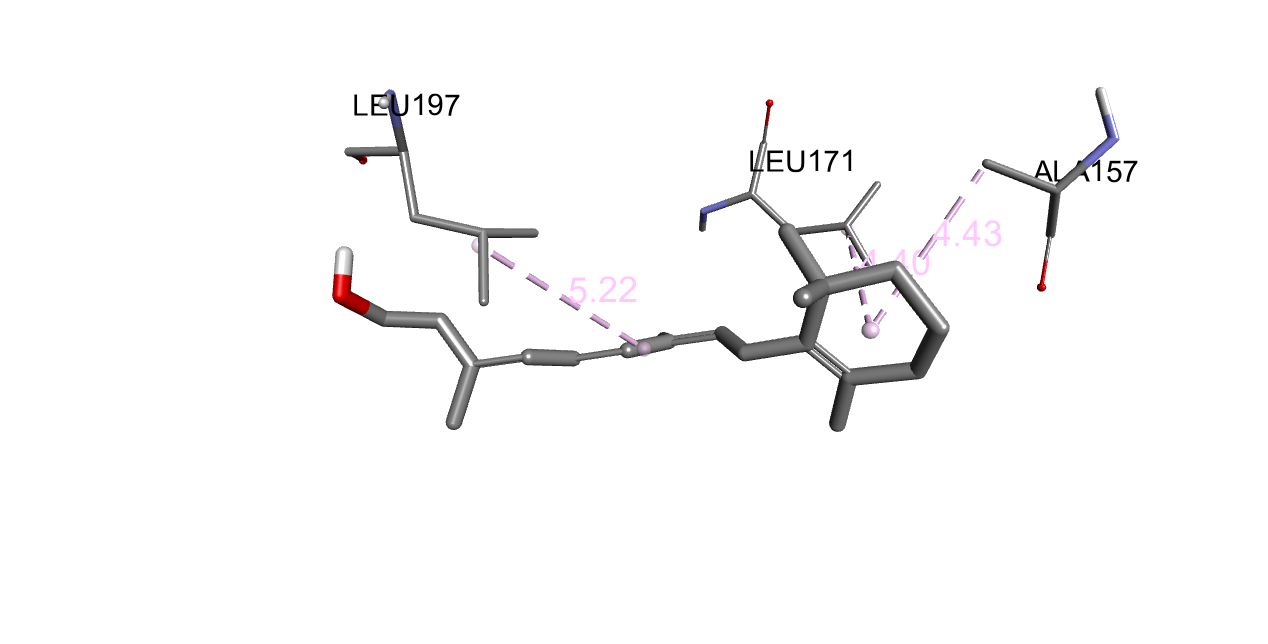

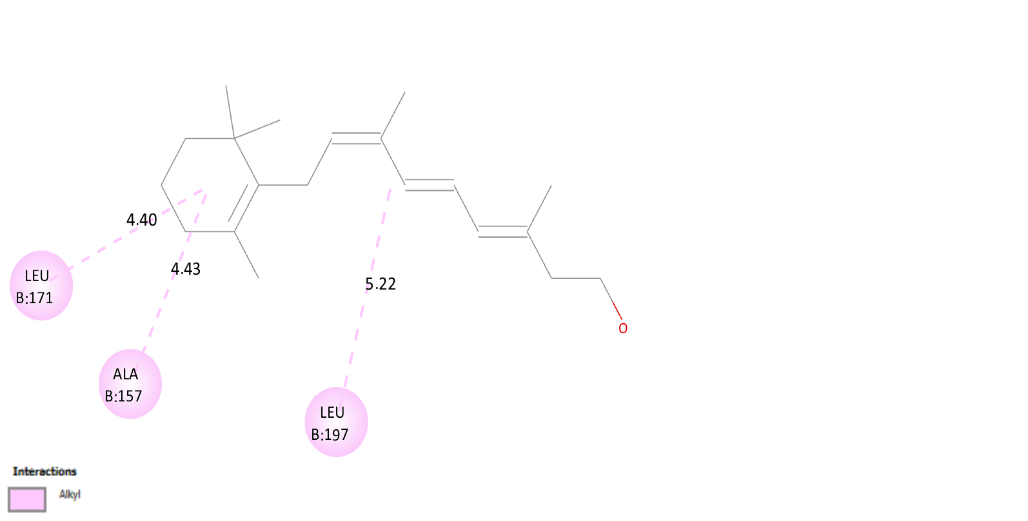


Figure S1: 2D and 3D interaction of Retinol with CYP450 2E1 (PDBID: 3T3Z).


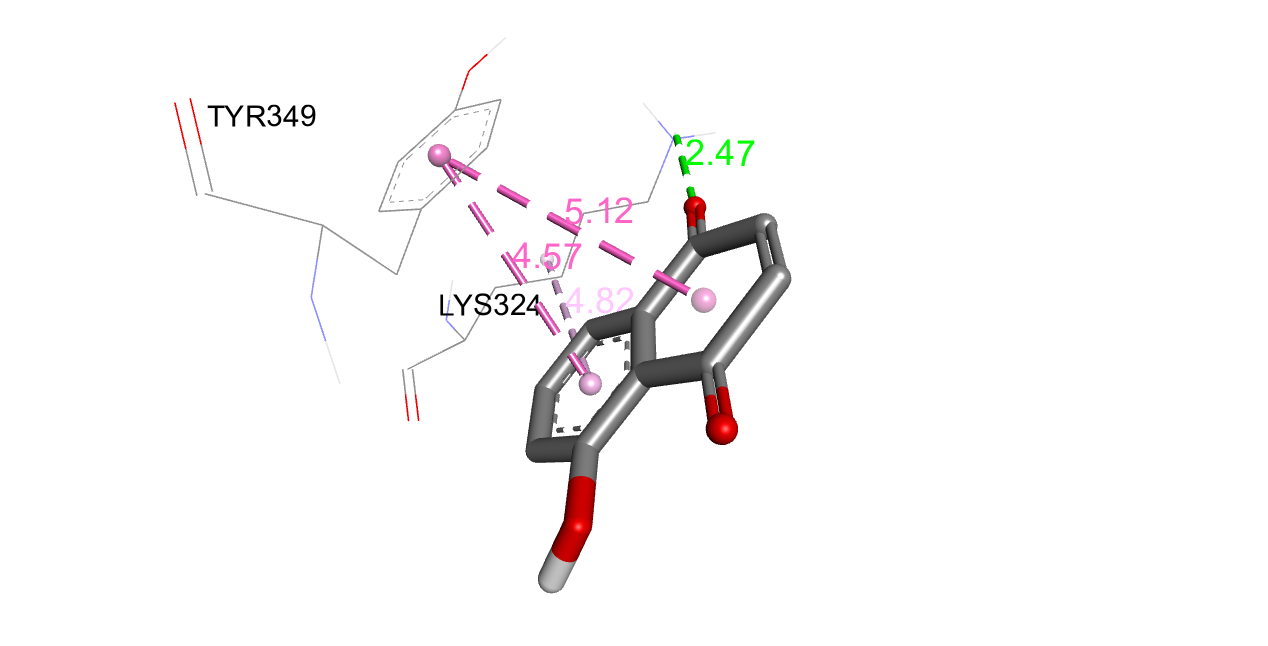

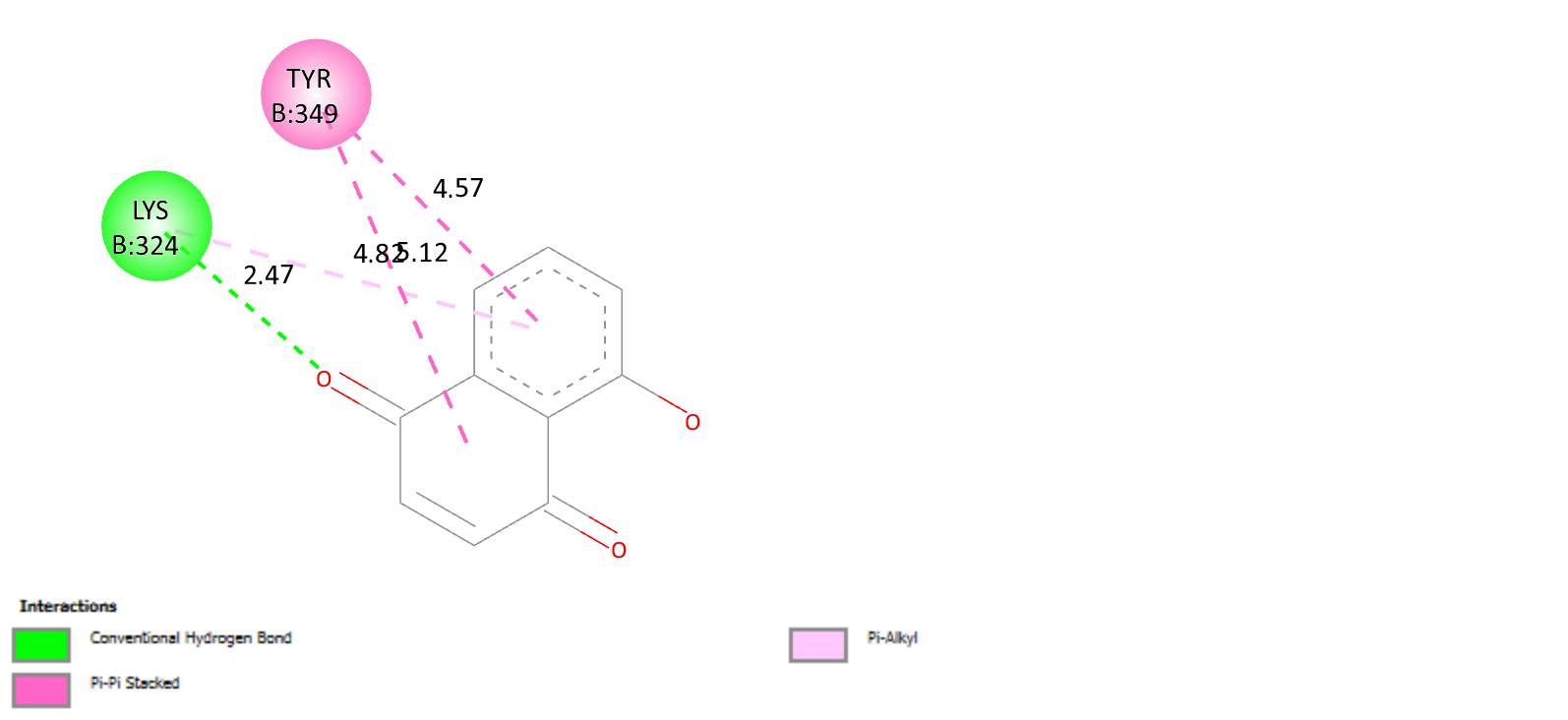


Figure S2: 2D and 3D interaction of Juglone with CYP450 2E1 (PDBID: 3T3Z).


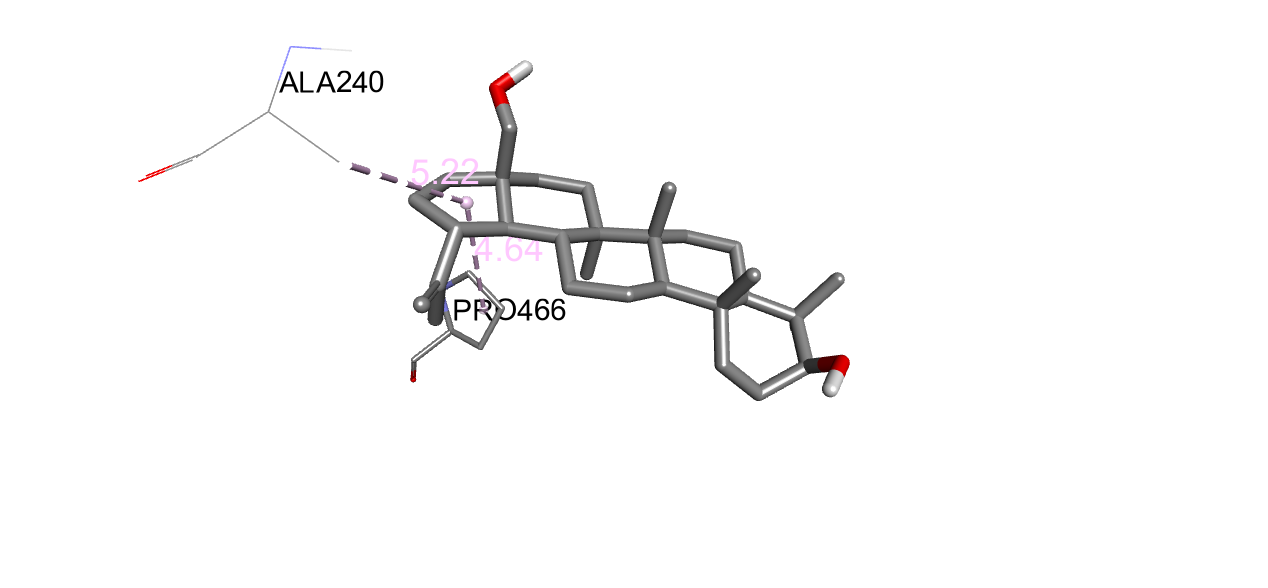

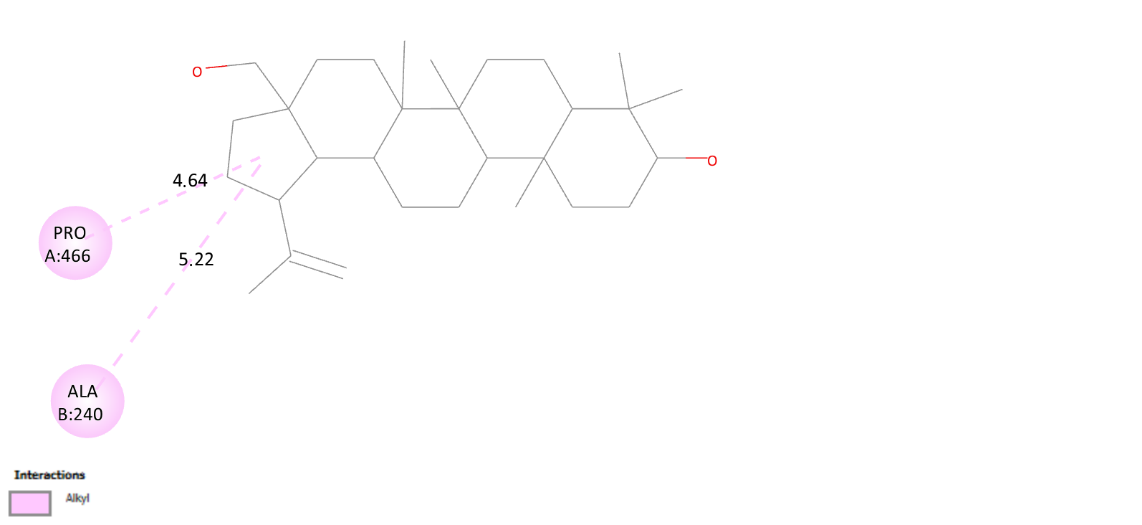


Figure S3: 2D and 3D interaction of Betulin with CYP450 2E1 (PDBID: 3T3Z).


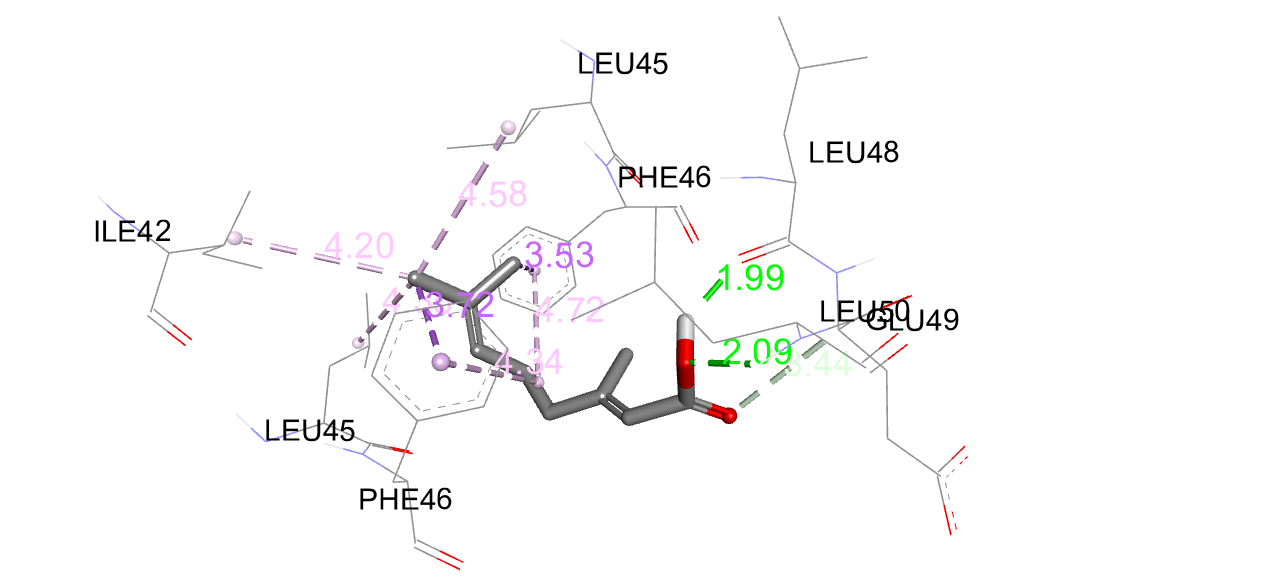

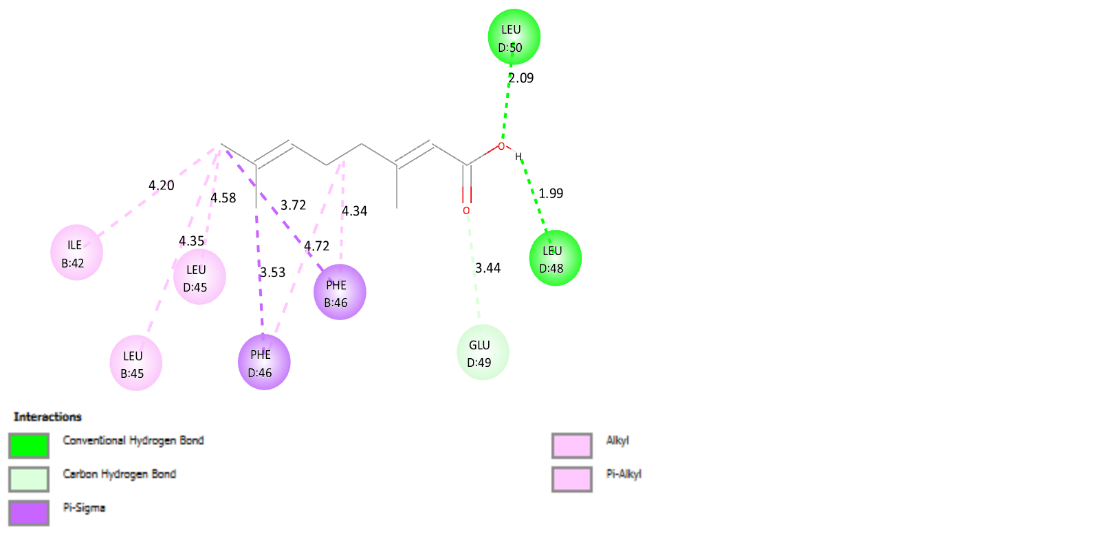


Figure S4: 2D and 3D interaction of Geranic Acidwith CYP450 2E1 (PDBID: 3T3Z).


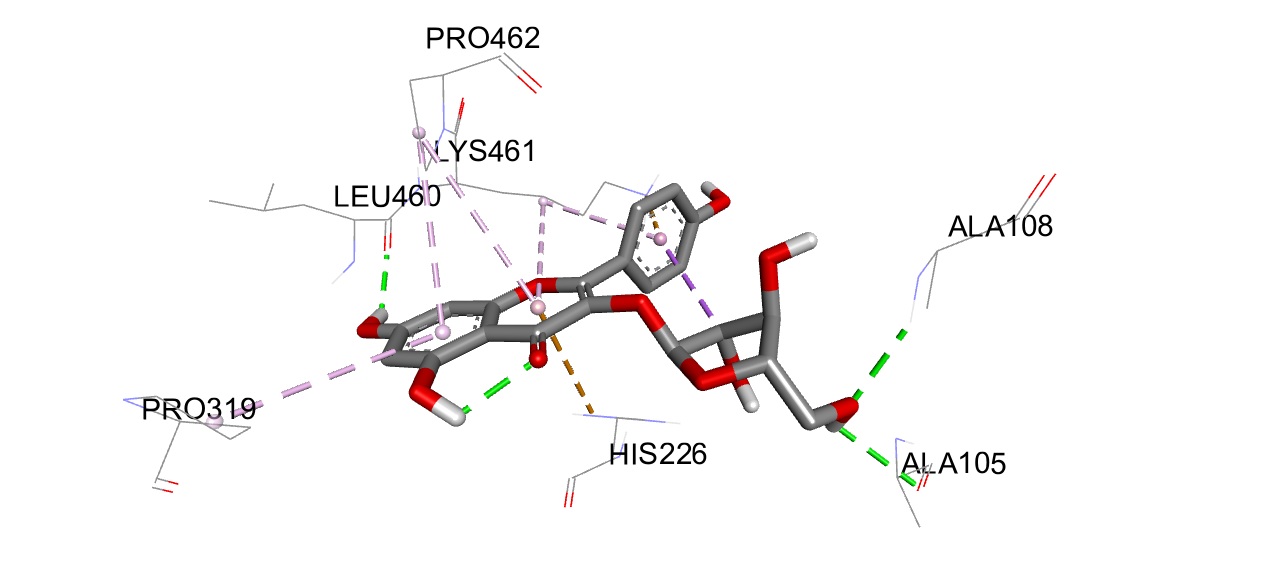

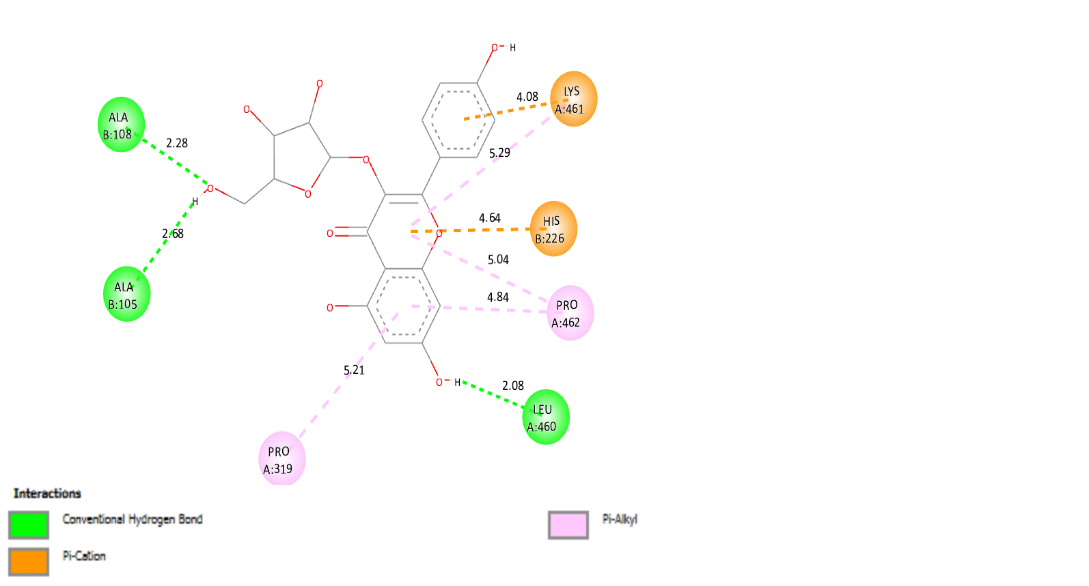


Figure S5: 2D and 3D interaction of Juglaninwith CYP450 2E1 (PDBID: 3T3Z).


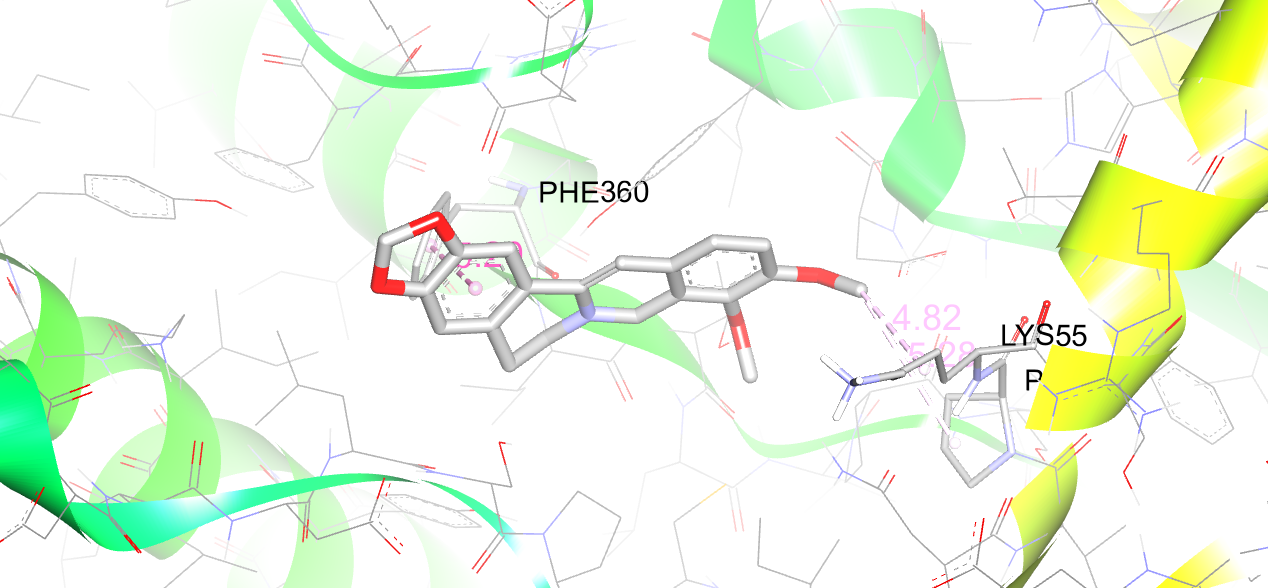

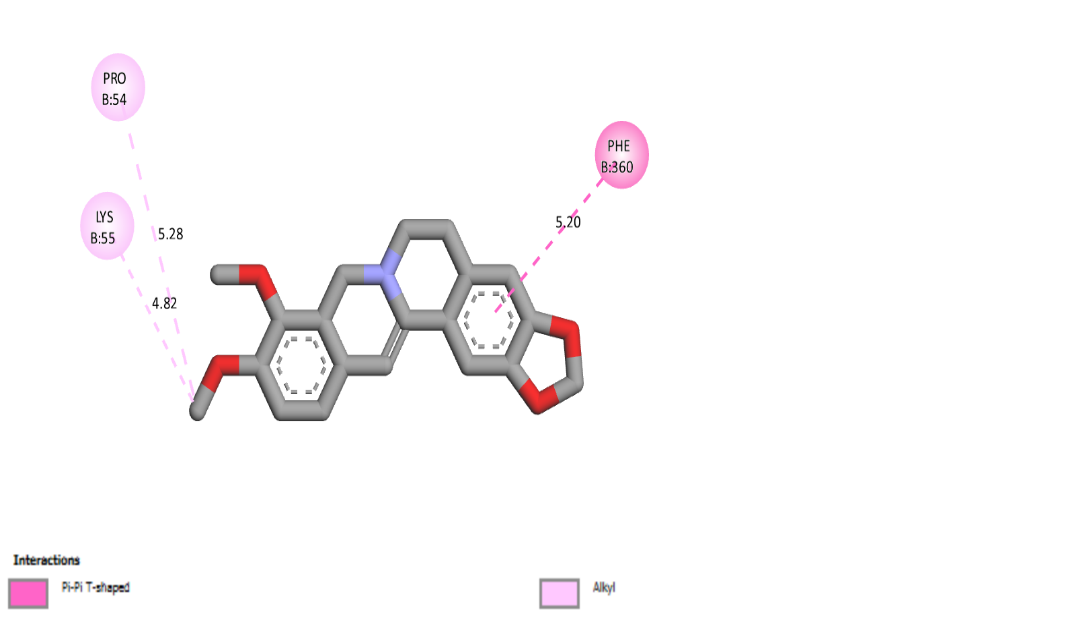


Figure S6: 2D and 3D interaction of Berberine with CYP450 2E1 (PDBID: 3T3Z).


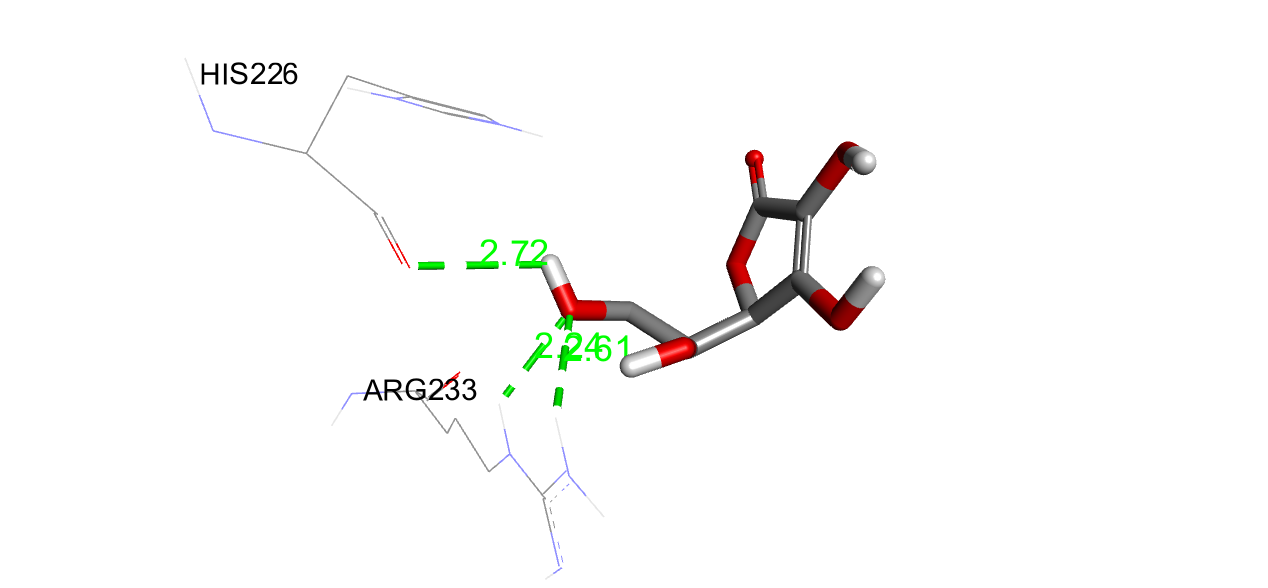

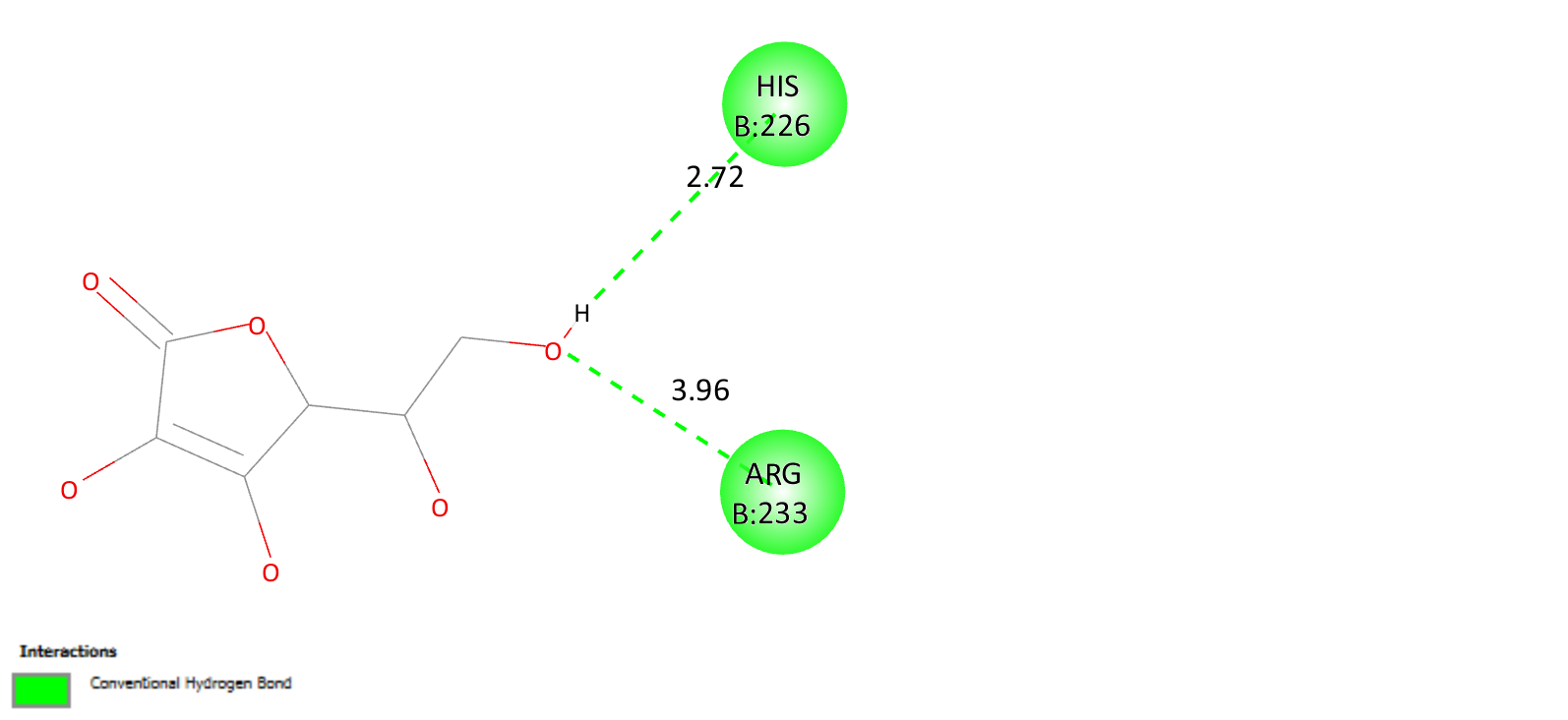


Figure S7: 2D and 3D interaction of Ascorbic acid with CYP450 2E1 (PDBID: 3T3Z).


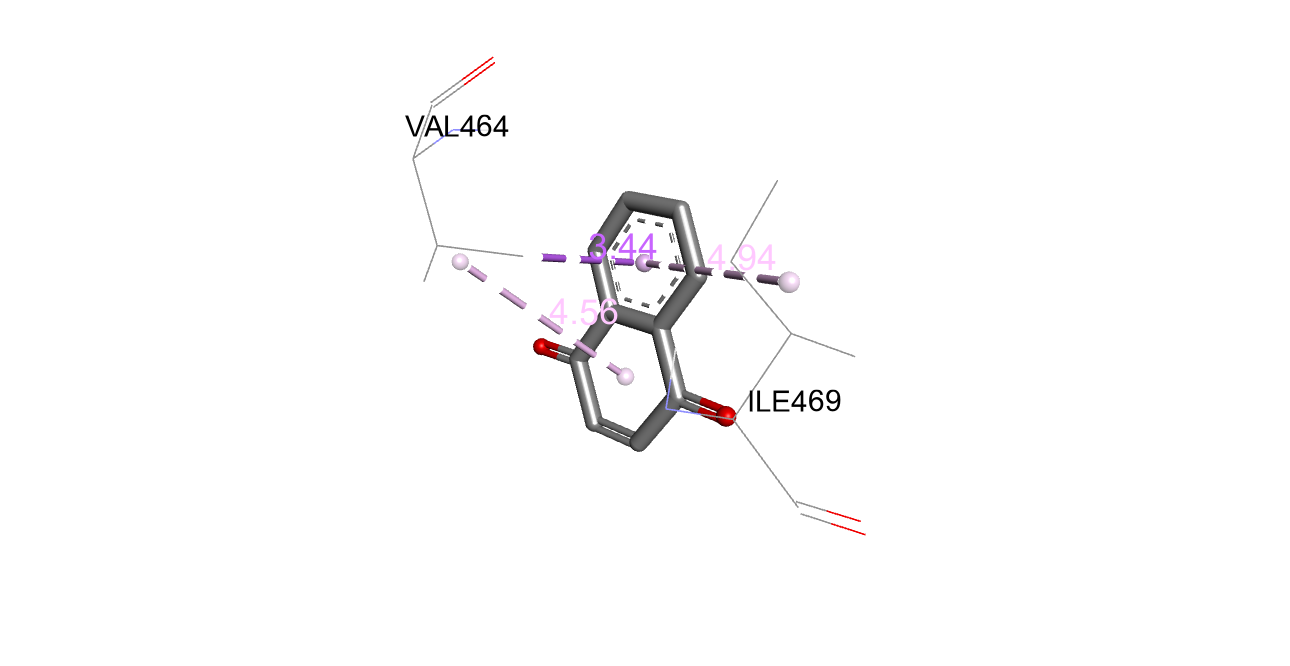


Figure S8: 3D interaction of 1,4-Naphthoquinonewith CYP450 2E1 (PDBID: 3T3Z).


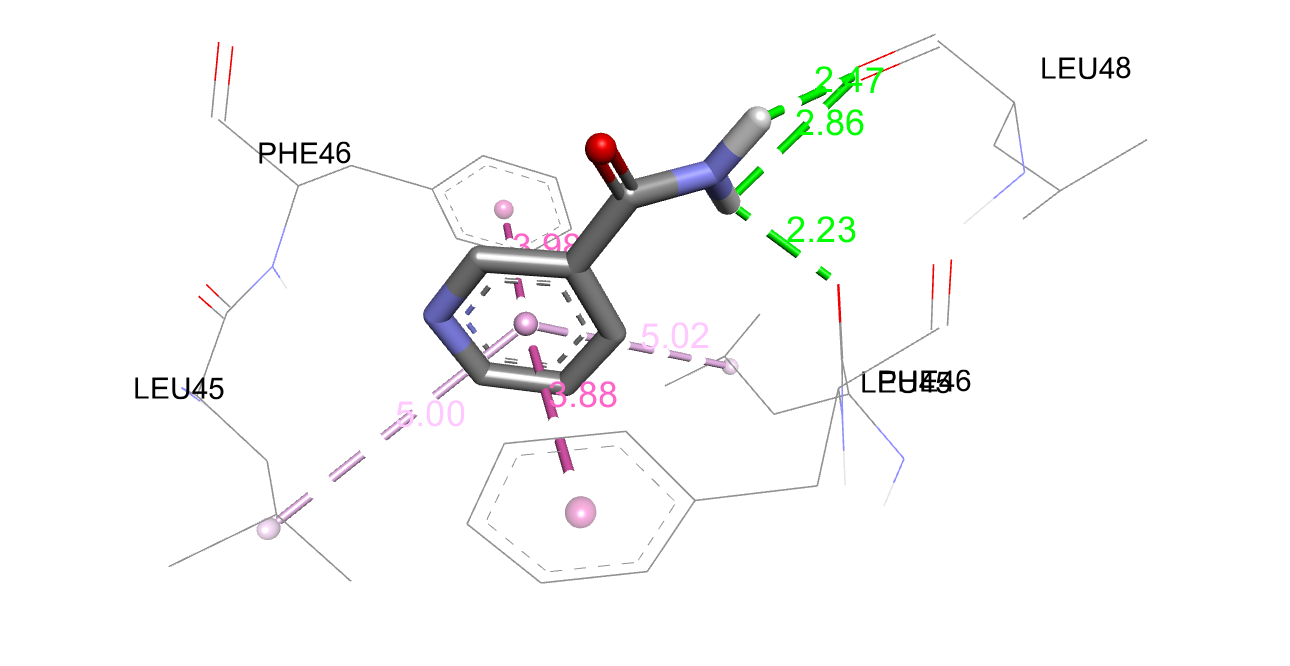

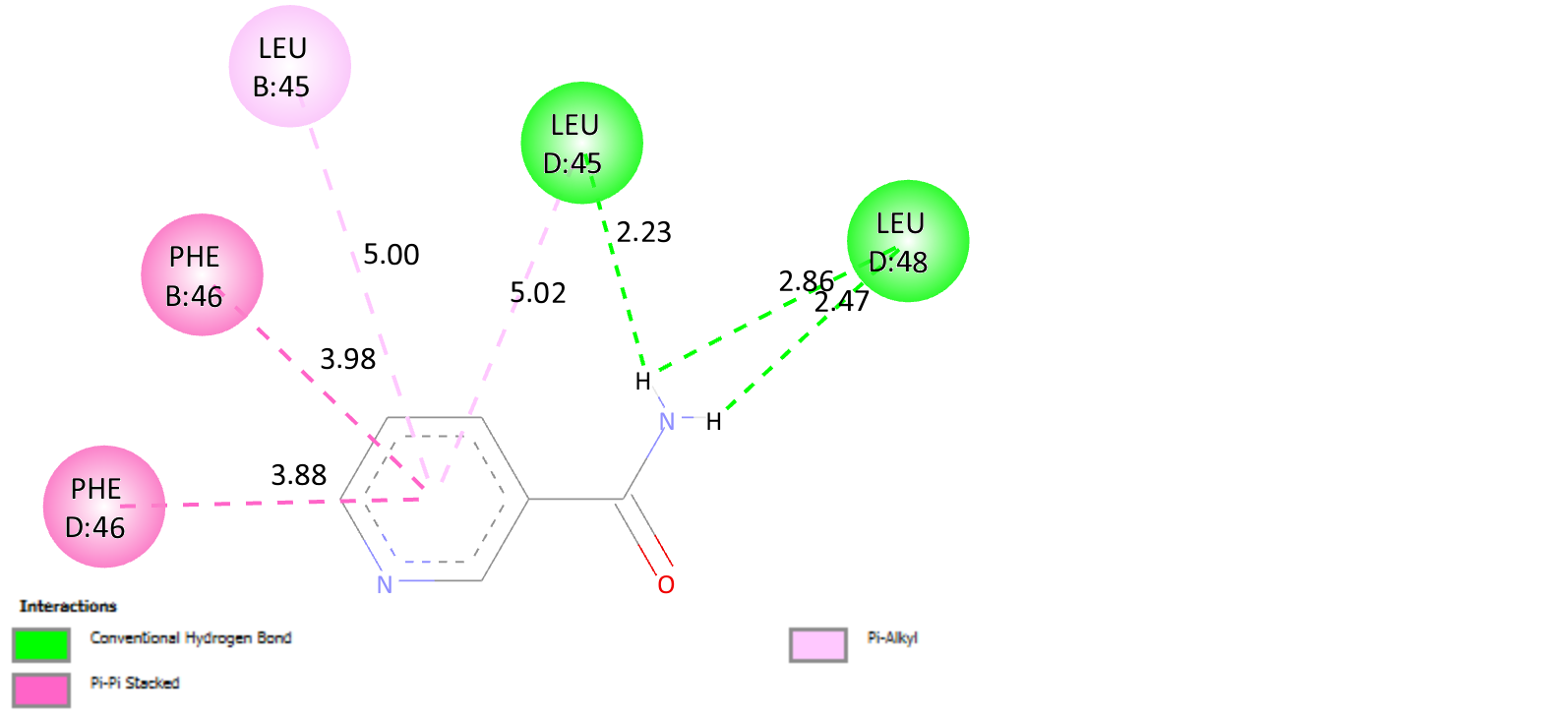


Figure S9: 2D and 3D interaction of Nicotinamidewith CYP450 2E1 (PDBID: 3T3Z).


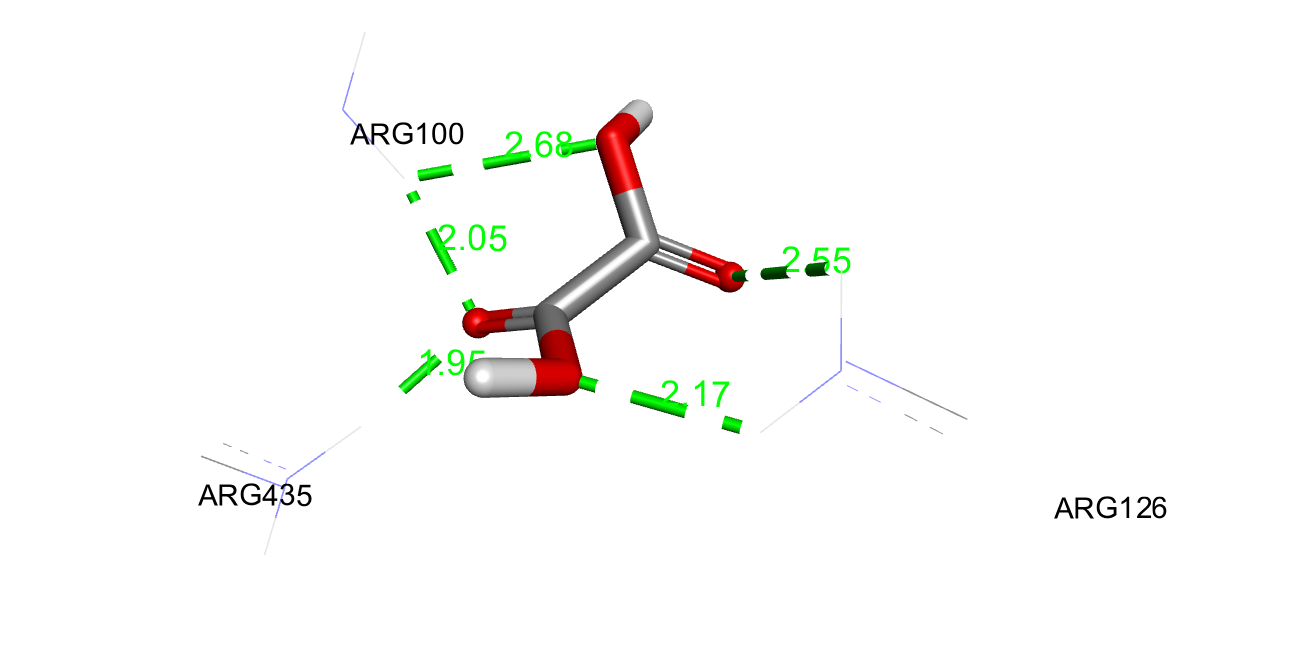

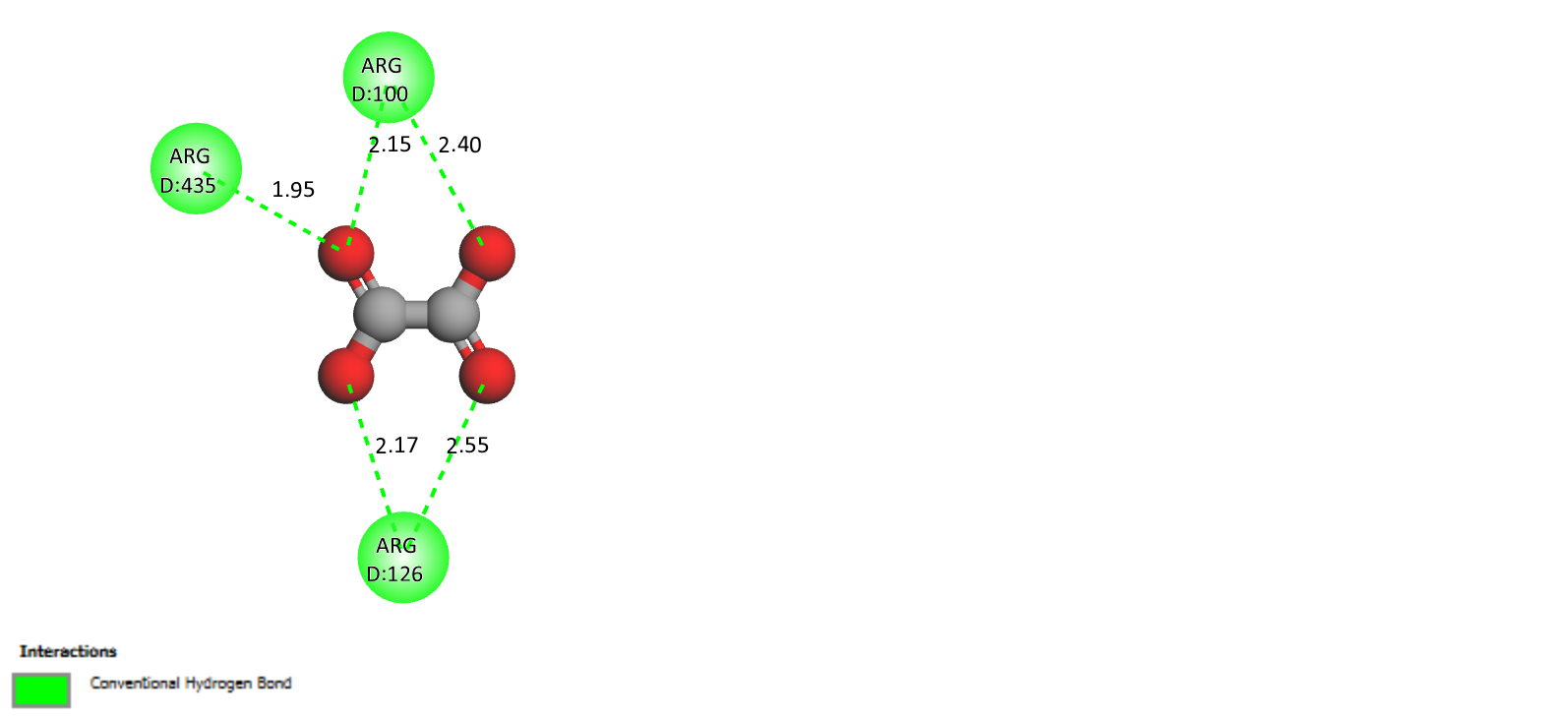


Figure S10: 2D and 3D interaction of Oxalic acid with CYP450 2E1 (PDBID: 3T3Z).


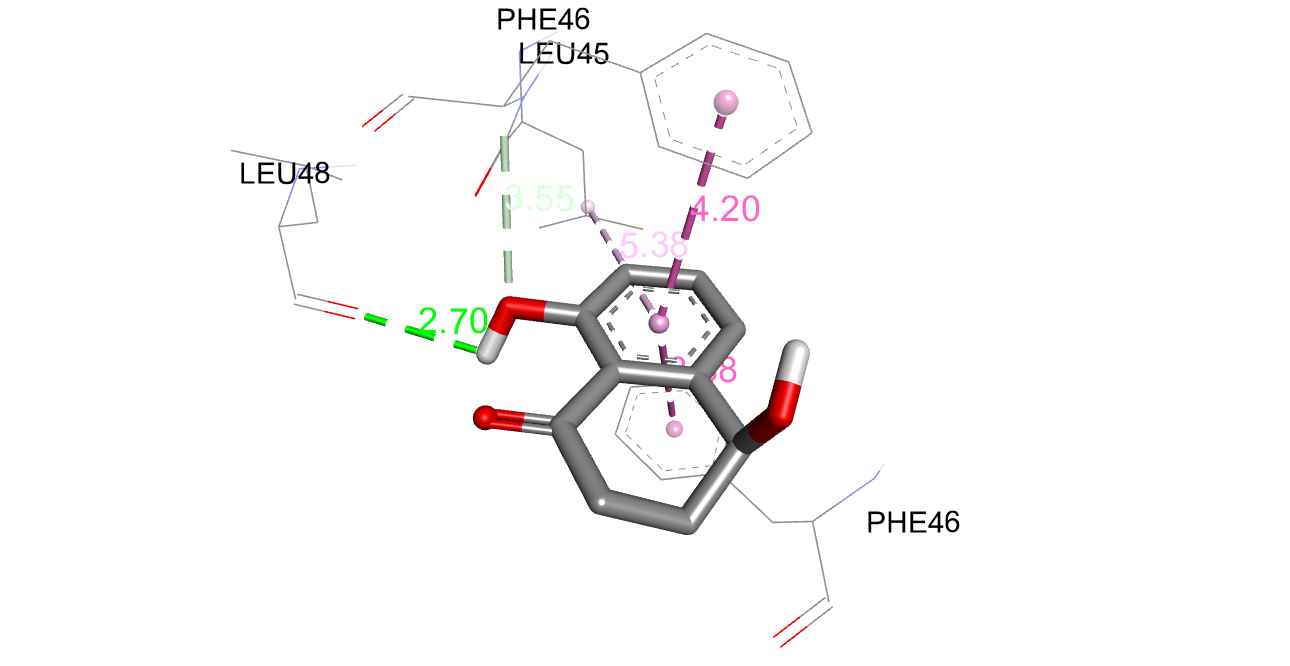

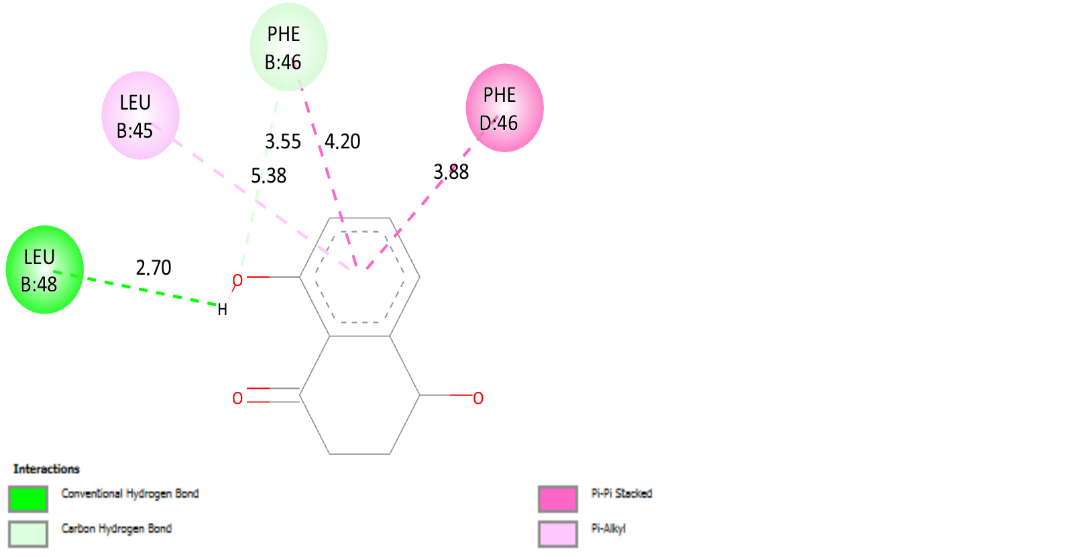


Figure S11: 2D and 3D interaction of (4S) 4,8-dihydroxytetralin-1-onewith CYP450 2E1 (PDBID: 3T3Z).


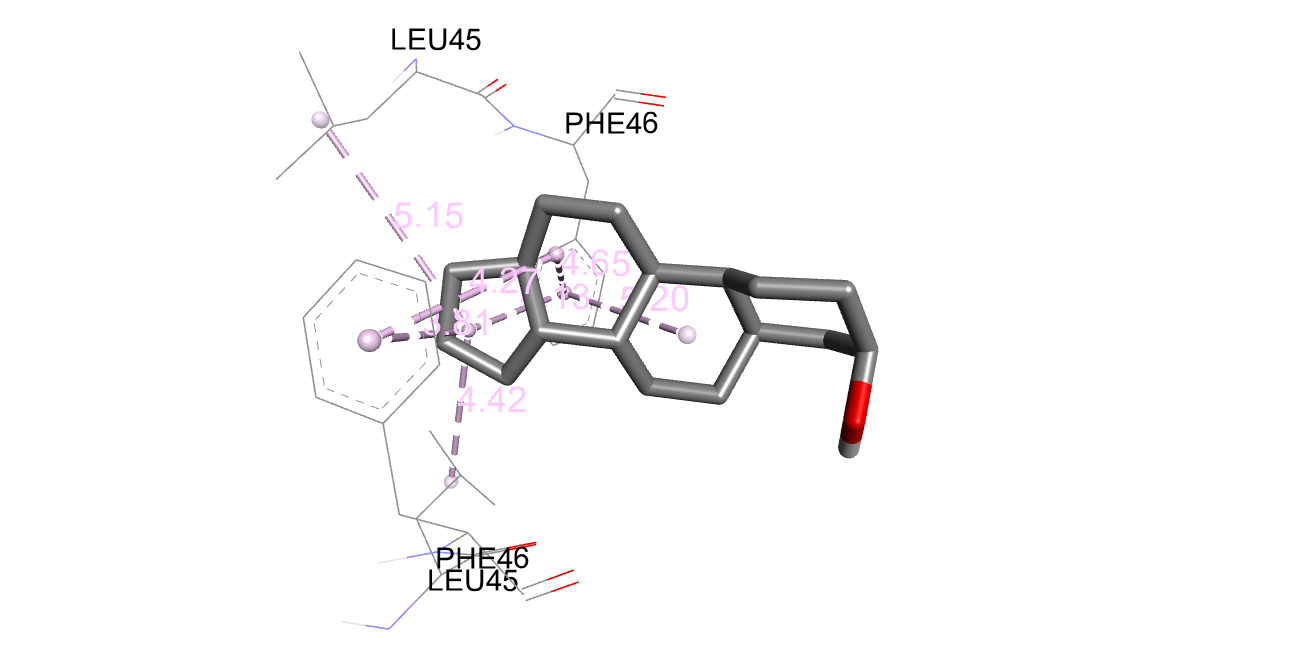

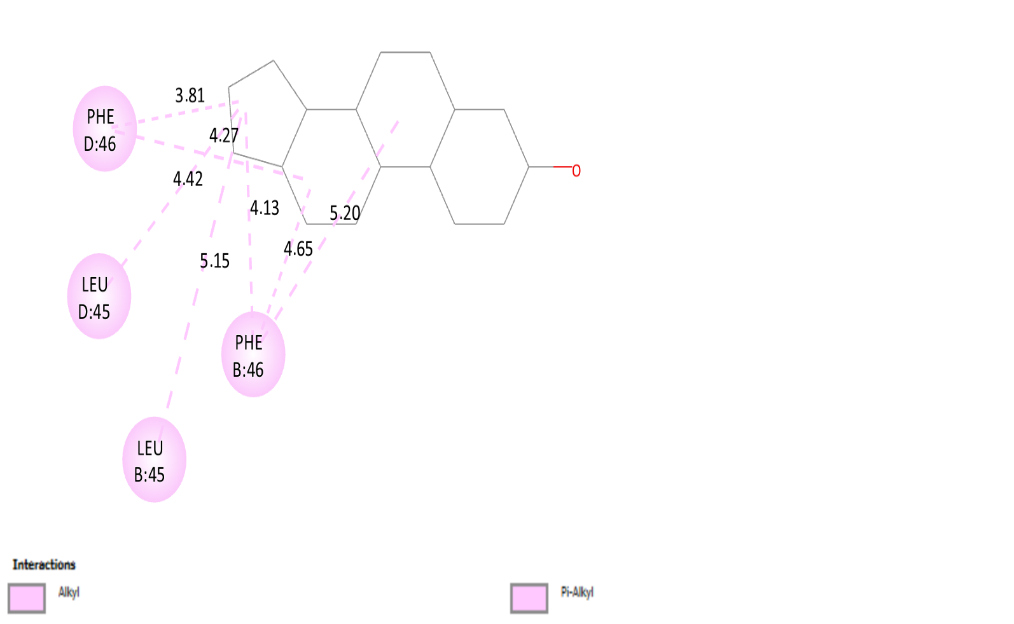


Figure S12: 2D and 3D interaction of Sterolwith CYP450 2E1 (PDBID: 3T3Z).


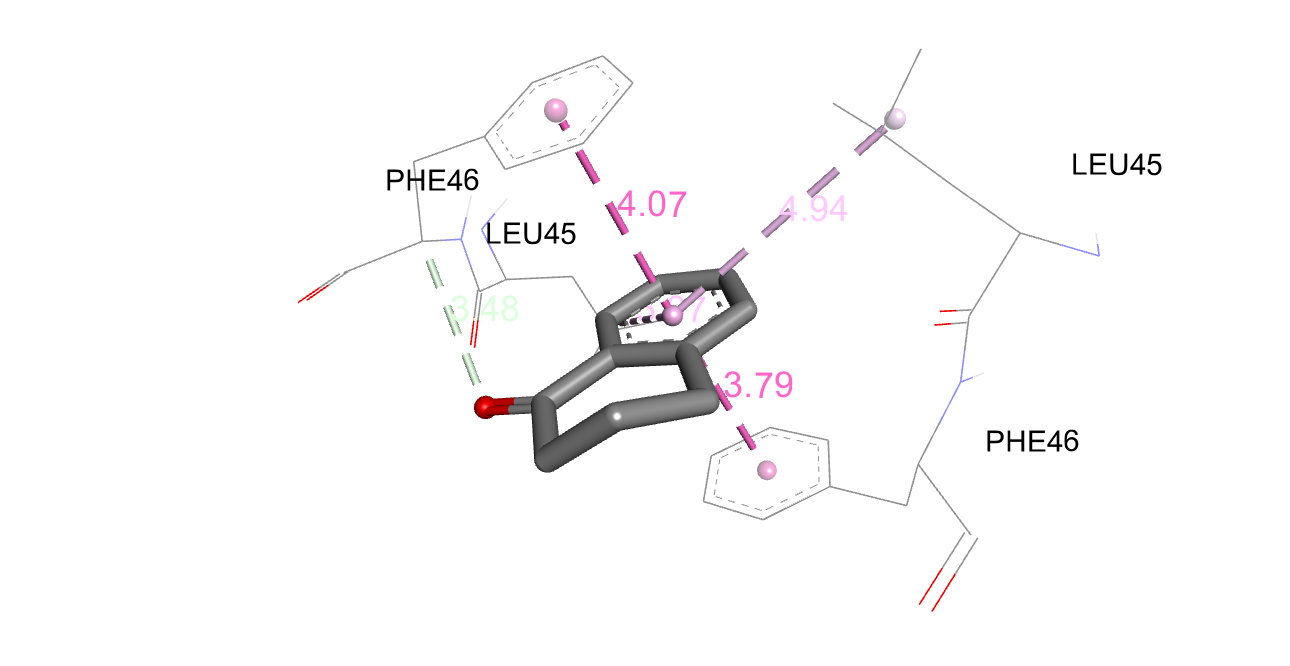


Figure S13: 3D interaction of 1-tetralonewith CYP450 2E1 (PDBID: 3T3Z).


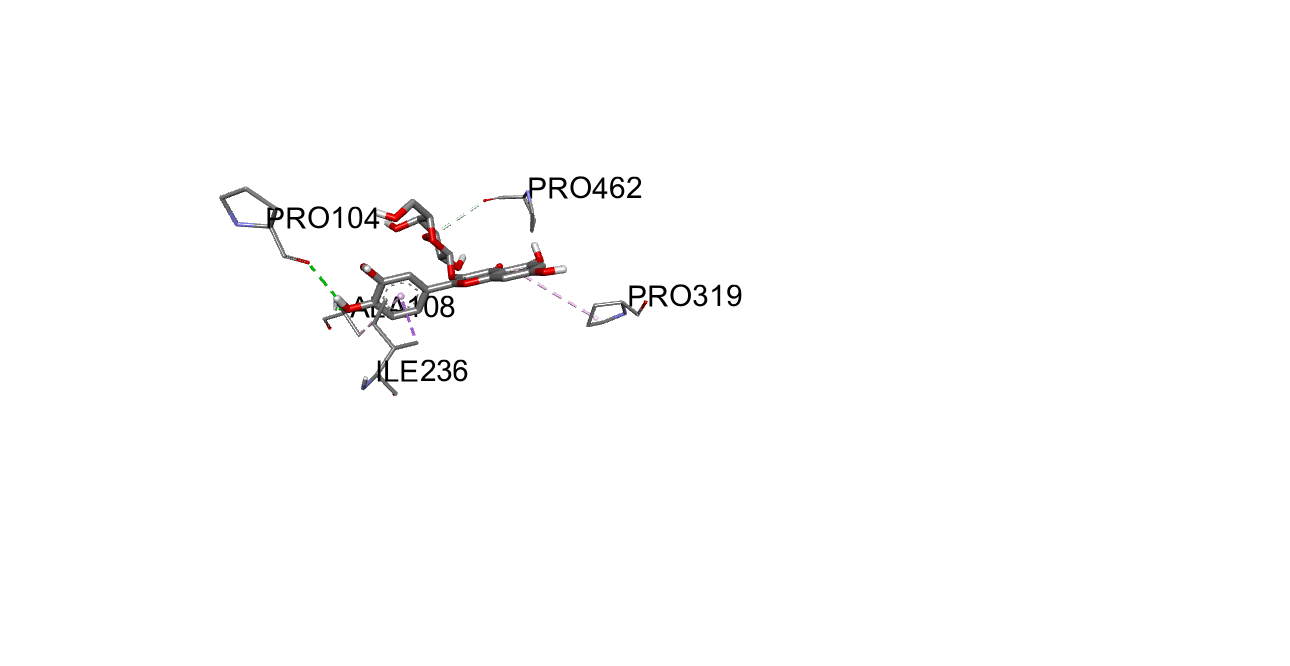

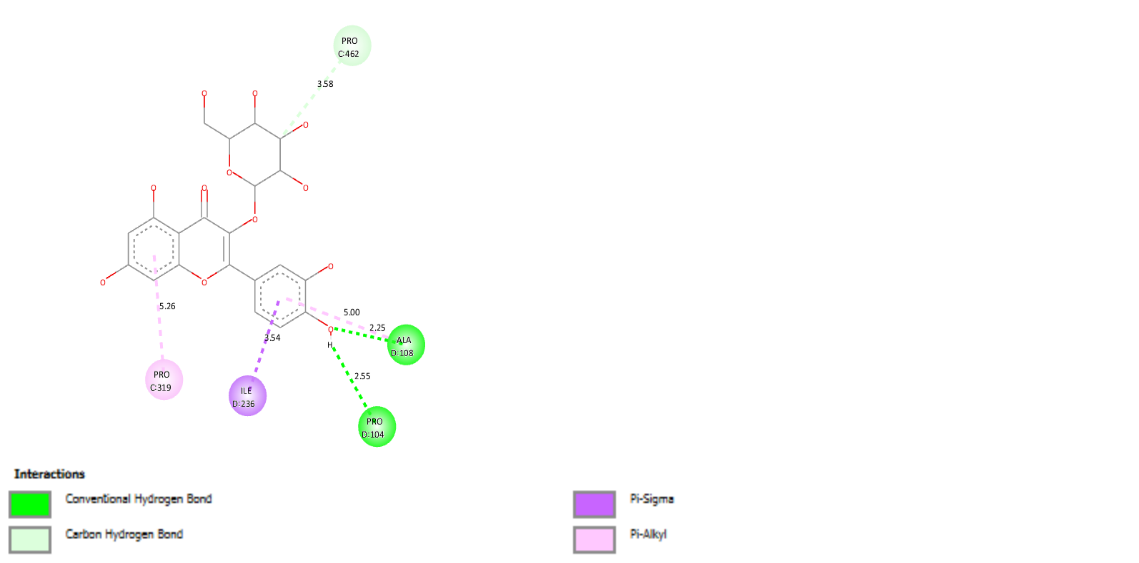


Figure S14: 2D and 3D interaction of Hyperoside with CYP450 2E1 (PDBID: 3T3Z).
